# Incidence, diagnosis, management and outcome of acute mesenteric ischaemia: a prospective, multicentre observational study (AMESI Study)

**DOI:** 10.1186/s13054-024-04807-4

**Published:** 2024-01-23

**Authors:** Annika Reintam Blaser, Merli Mändul, Martin Björck, Stefan Acosta, Miklosh Bala, Zsolt Bodnar, Dumitru Casian, Zaza Demetrashvili, Mario D’Oria, Virginia Durán Muñoz-Cruzado, Alastair Forbes, Hanne Fuglseth, Moran Hellerman Itzhaki, Benjamin Hess, Karri Kase, Mikhail Kirov, Kristoffer Lein, Matthias Lindner, Cecilia Inés Loudet, Damian J. Mole, Marko Murruste, Alexandre Nuzzo, Sten Saar, Maximilian Scheiterle, Joel Starkopf, Peep Talving, Anna-Liisa Voomets, Kenneth K. T. Voon, Mohammad Alif Yunus, Kadri Tamme, AMESI Investigators (Collaborators), Olivier Corcos, Yves Castier, Maxime Ronot, Alan Biloslavo, Lucia Paiano, Gunnar Elke, Denise Nagel, David I. Radke, Jacqueline Vilca Becerra, María Elina Abeleyra, Martin Cahenzli, Tatjana Semenkova, Anton Nikonov, Alexey Smetkin, Geir Ivar Nedredal, Øivind Irtun, Oded Cohen-Arazi, Asaf Keda, Gheorghe Rojnoveanu, Alexandr Ursu, Felipe Pareja Ciuró, Anabel García-Leon, Carlos Javier García-Sánchez, Lim Jia Hui, Loy Yuan Ling, Ilya Kagan, Pierre Singer, Edgar Lipping, Ana Tvaladze, Dimitrios Damaskos, Darja Clinch, Too Xiao Qing, Morten Vetrhus, Jacopo Martellucci, Giulia Cerino, Donghuang Hong, Jinsheng Liu, Ernest Ong, Kursat Kundogan, Tutkun Talih, Lovenish Bains, Diego Visconti, Lorenzo Gibello, Ruhi Fadzlyana Jailani, Muhammad Amirul Ashra, Andee Dzulkarnaen Zakaria, Ahmad Faiz Najmuddin Mohd Ghazi, Nur Suriyana Abd Ghani, Mohd Fadliyazid Ab Rahim, Goran Augustin, Damir Halužan, Mohan Gurjar, Rahul Rahul, Firdaus Hayati, Jin-Jiun Mah

**Affiliations:** 1 Institute of Clinical Medicine, University of Tartu, Puusepa 8, 50406 Tartu, Estonia; 2 Department of Intensive Care Medicine, Lucerne Cantonal Hospital, Lucerne, Switzerland; 3 Institute of Mathematics and Statistics, University of Tartu, Tartu, Estonia; 4 Estonian Genome Center, Institute of Genomics, University of Tartu, Tartu, Estonia; 5 Department of Surgical Sciences, Section of Vascular Surgery, Uppsala University, Uppsala, Sweden; 6 Department of Clinical Sciences, Lund University, Malmö, Sweden; 7 Hadassah Medical Center and Faculty of Medicine, Hebrew University of Jerusalem, Jerusalem, Israel; 8 Letterkenny University Hospital, Letterkenny, Ireland; 9 University Clinic of Vascular Surgery, “Nicolae Testemitanu” State University of Medicine and Pharmacy of the Republic of Moldova, Chişinău, Moldova; 10 N. Kipshidze Central University Hospital, Tbilisi, Georgia; 11 University Hospital of Trieste ASUGI, Trieste, Italy; 12 Virgen del Rocío University Hospital, Seville, Spain; 13 Department of Gastrointestinal Surgery, Stavanger University Hospital, Stavanger, Norway; 14 Intensive Care Unit and Institute for Nutrition Research, Rabin Medical Center, University of Tel Aviv, Petah Tikva, Israel; 15 Tartu University Hospital, Puusepa 8, Tartu, Estonia; 16 Department of Anesthesiology and Intensive Care Medicine, Northern State Medical University and City Hospital #1, Arkhangelsk, Russia; 17 University Hospital North Norway and UiT The Arctic University of Norway, Tromsö, Norway; 18 Universitätsklinikum Schleswig-Holstein, Campus Kiel, Kiel, Germany; 19 Hospital General San Martin de La Plata, Buenos Aires, Argentina; 20 Chair of Surgery, University of Edinburgh Centre for Inflammation Research, Royal Infirmary of Edinburgh, Edinburgh, UK; 21 Intestinal Stroke Center, Department of Gastroenterology, IBD and Intestinal Failure, AP-HP. Nord, Beaujon Hospital, Paris Cité University, Paris, France; 22 Division of Acute Care Surgery, North Estonia Medical Centre, Tallinn, Estonia; 23 Azienda Ospedaliera Universitaria Careggi, Florence, Italy; 24 Colorectal Surgery, Sarawak General Hospital, Kuching, Malaysia; 25 General Surgeon of General Surgery Department, Hospital Melaka, Malacca, Malaysia

**Keywords:** Mesenteric ischaemia, Epidemiology, Diagnosis, Management, Outcome

## Abstract

**Background:**

The aim of this multicentre prospective observational study was to identify the incidence, patient characteristics, diagnostic pathway, management and outcome of acute mesenteric ischaemia (AMI).

**Methods:**

All adult patients with clinical suspicion of AMI admitted or transferred to 32 participating hospitals from 06.06.2022 to 05.04.2023 were included. Participants who were subsequently shown not to have AMI or had localized intestinal gangrene due to strangulating bowel obstruction had only baseline and outcome data collected.

**Results:**

AMI occurred in 0.038% of adult admissions in participating acute care hospitals worldwide. From a total of 705 included patients, 418 patients had confirmed AMI. In 69% AMI was the primary reason for admission, while in 31% AMI occurred after having been admitted with another diagnosis. Median time from onset of symptoms to hospital admission in patients admitted due to AMI was 24 h (interquartile range 9-48h) and time from admission to diagnosis was 6h (1–12 h). Occlusive arterial AMI was diagnosed in 231 (55.3%), venous in 73 (17.5%), non-occlusive (NOMI) in 55 (13.2%), other type in 11 (2.6%) and the subtype could not be classified in 48 (11.5%) patients. Surgery was the initial management in 242 (58%) patients, of which 59 (24.4%) underwent revascularization. Endovascular revascularization alone was carried out in 54 (13%), conservative treatment in 76 (18%) and palliative care in 46 (11%) patients. From patients with occlusive arterial AMI, revascularization was undertaken in 104 (45%), with 40 (38%) of them in one site admitting selected patients. Overall in-hospital and 90-day mortality of AMI was 49% and 53.3%, respectively, and among subtypes was lowest for venous AMI (13.7% and 16.4%) and highest for NOMI (72.7% and 74.5%). There was a high variability between participating sites for most variables studied.

**Conclusions:**

The overall incidence of AMI and AMI subtypes varies worldwide, and case ascertainment is challenging. Pre-hospital delay in presentation was greater than delays after arriving at hospital. Surgery without revascularization was the most common management approach. Nearly half of the patients with AMI died during their index hospitalization. Together, these findings suggest a need for greater awareness of AMI, and better guidance in diagnosis and management.

*Trial registration*: NCT05218863 (registered 19.01.2022).

**Supplementary Information:**

The online version contains supplementary material available at 10.1186/s13054-024-04807-4.

## Background

Acute mesenteric ischaemia (AMI) occurs infrequently and is difficult to diagnose due to non-specific symptoms and the absence of well-established diagnostic biomarkers. Consequently, AMI is insufficiently studied and lacks standardized management internationally. Most available evidence originates from retrospective single-centre studies with a long duration of data collection, indicating that AMI has a very high lethality with only modest improvement in outcomes over recent decades [1]. In a systematic review and a recent population-based retrospective study, AMI was the primary diagnosis in approximately 5–7 patients per 10,000 hospital admissions [1, 2], but the incidence is likely underestimated due to poor recognition, and the true worldwide incidence is not known. Heterogeneous clinical manifestations and pathophysiological mechanisms of different subtypes of AMI (occlusive arterial or venous, non-occlusive) usually result in multiple specialties being involved in the primary diagnosis and management of AMI [3–7], and key similarities and differences between subtypes of AMI are incompletely studied. Regardless of the specific approaches to diagnosis and management for different subtypes of AMI, all subtypes ultimately lead to severe consequences and high mortality [1, 2]. A recent survey has identified delay in diagnosis of AMI and heterogeneity in management approaches as contributing to poor outcomes [8]. Recent guidelines accentuate the importance of computed tomography angiography being performed in all patients with suspected AMI, immediate surgical treatment in patients with overt peritonitis, and emphasize revascularization in cases of occlusive arterial AMI, whereas there is more uncertainty regarding other recommendations [9, 10].

Due to the rare occurrence, multifaceted nature and diverse medical specialties involved in the management, the patterns of diagnosis, differentiation between subtypes and management of AMI have not been studied in a prospective multicentre design.

The aim of this multicentre prospective observational study (NCT05218863) was to identify the incidence of AMI among patients admitted to hospital and to describe patient characteristics, diagnostics, management and outcomes of AMI and its different subtypes.

## Methods

### Study design

All adult patients admitted or transferred to participating hospitals during a 10-month period (06.06.2022–05.04.2023) were screened (excluding long-term chronic care, paediatric and psychiatry wards) for suspected or confirmed mesenteric ischaemia. Suspicion of AMI was based on routine clinical assessment at each site. All sites were provided materials to instruct the wards and encouraged to use electronic patient data management systems and radiology databases to identify eligible patients. Specific guidance for diagnosis and management of AMI was not provided to avoid interference with usual clinical practice.

All patients with suspicion of or confirmed AMI due to any mechanism were included. If suspicion of AMI was not confirmed or strangulating bowel obstruction (SBO) with local intestinal gangrene was the final diagnosis, only baseline data and hospital mortality outcome were collected. Patients with extensive bowel ischaemia (as determined by the local investigator) due to SBO were included as “confirmed AMI due to other specific mechanism”.

If the diagnosis of AMI was confirmed, a comprehensive data collection, including diagnostics, management, hospital outcomes and 90-day survival, was performed.

*Exclusion criteria* were age < 18 years; consent declined by patient or next of kin; and chronic mesenteric ischaemia without an acute event.

The study complied with the Strengthening the Reporting of Observational studies in Epidemiology (STROBE) statement for cohort studies (Additional file 1: Table S1).

### Study objectives

#### Primary objective

- To identify the incidence of AMI and its different subtypes in hospitalized adult patients.

#### Secondary objectives

- To clarify the differences in patient characteristics at baseline (demographic, clinical and laboratory data at the time point of suspicion of AMI) and outcomes in different subtypes of AMI.
- To compare patient characteristics at baseline, and mortality in confirmed AMI vs suspected but not confirmed AMI.
- To identify key factors associated with delays in the process of care from onset of symptoms to ultimate management of AMI in its different subtypes.
- To identify the time from onset of symptoms and from hospital admission to diagnosis of AMI in its different subtypes.

#### Tertiary objectives

- To describe patterns and pathways of reaching a diagnosis of AMI and its different subtypes.
- To describe management of AMI in its different subtypes.
- To describe the decision-making process (which management options were available, which were discussed within the clinical team and which with patient/family).

### Definitions

*Acute mesenteric ischaemia* (AMI) was defined as the occurrence of an abrupt cessation of the mesenteric blood flow with an acute onset of symptoms [10].

Subtypes of AMI were defined as follows:

1. *Occlusive intestinal ischaemia*: Decreased mesenteric blood flow due to acute thromboembolic high-grade stenosis or occlusion of mesenteric vessels with further subdivision:
  arterial embolism
  arterial thrombosis
  venous thrombosis
2. *Non-occlusive intestinal ischaemia* (NOMI): Acute severe ischaemia of the intestine developing without an acute thromboembolic high-grade stenosis or occlusion.
3. *Intestinal ischaemia in specific conditions or* via *unclear mechanisms*:
  AMI due to abdominal compartment syndrome [11]
  AMI after abdominal aortic aneurysm repair
  AMI due to aortic dissection
  AMI in patients with intra-aortic balloon counterpulsation or another mechanical cardiac support device
  Acute-on chronic mesenteric ischaemia (chronic mesenteric ischaemia that led to emergency admission due to an acute ischaemic event with intestinal infarction).
  Intestinal infarction due to any other cause or an unclear mechanism

*Suspicion of AMI* was raised based on a clinical decision by local investigators, including general guidance such as the following: abdominal pain (usually diffuse and strong) without an obvious non-AMI diagnosis, or critically ill patients with suspicion of NOMI.

*Confirmation of AMI* was verified by one or more of the following: CT-scan, mesenteric angiography, endoscopy, surgery, histology, autopsy.

*Local intestinal gangrene due to SBO* comprised a separate group and was documented with minimal data collection (baseline data and hospital survival) similarly to suspected AMI. The following guidance was given for screening of patients with SBO:

- Intestinal obstruction due to adhesions with small or large bowel strangulation
- Incarcerated hernia with small or large bowel strangulation
- Small or large bowel volvulus

The rationale to prospectively create a separate group for SBO, considered an important differential diagnosis of AMI [12, 13], was to address the expected difficulty in categorizing patients with local intestinal gangrene due to a mechanical cause.

*Chronic mesenteric ischaemia (CMI)* was defined as ischaemic symptoms caused by insufficient blood supply to the gastrointestinal tract with a duration of at least 3 months [10]. Typical presentation includes postprandial pain, weight loss resulting from fear of eating or unexplained diarrhoea.

*Acute ischaemic event* in a patient with known or suspected CMI (acute-on-chronic mesenteric ischaemia) refers to acute onset of more severe symptoms of mesenteric ischaemia necessitating hospitalization. These patients were included in the study.

*Acute care hospital admissions* include all adult patients receiving active in-hospital treatment for an injury or episode of illness, including any medical condition and any surgery (patients after elective surgery need active care during recovery from surgery). Patients admitted to psychiatry or chronic/long-term care wards were excluded to retrieve this total number of acute care admissions.

### Ethics

Primary ethical approval was obtained from the Ethics Committee of the University of Tartu (357/T-8 and 364M-7). Each participating site obtained local Ethics Committee approval according to site, country and institutional regulations. Delayed informed consent was obtained from the patient or patient’s next of kin/proxy at the first possibility if requested by the local ethics committee. Patients were excluded from the study and any already collected data was deleted if the patient or the patient’s next of kin later declined participation in the study. Participation in the study did not influence any medical decisions, only data on provided medical care as usual in participating hospitals and resulting outcomes were collected.

Data were recorded in an electronic Case Report Form (using a REDCap platform) in a pseudonymized way and stored on a secure server of the University of Tartu.

### Sample size calculation

A group size of at least *n* = 40 patients for each subtype of AMI was considered necessary to adequately describe the incidence, outcome, diagnosis and management. Based on previous studies [1, 2], it was estimated that the proportion of the least frequent form of AMI is around 10% of all AMI cases. Accordingly, the aim was to include at least 400 patients with confirmed AMI. We estimated that 0.06% (0.05–0.07) of all adult patients hospitalized in acute care hospitals have AMI [1, 2]. Accordingly, a total of about 666,000 hospital admissions would need to be screened to identify these 400 patients. Based on that, 33 hospitals with a mean yearly case load of 40,000 patients each recruiting patients for 6 months would be needed. Considering a possibly lower incidence in some countries/hospitals and patients in whom informed consent is declined, the aim was to recruit 40 hospitals to reach our final target within 6 months. It was not possible to estimate the number of cases with suspected but not confirmed AMI. A planned interim analysis was performed at 4 months, and thereafter, it was decided to prolong the study duration to 10 months to reach the target of 400 patients.

### Statistics

Data are presented as number and proportions (%), or medians with interquartile ranges. Normality was assessed by the Kolmogorov–Smirnov test. Descriptive statistics were used to describe the incidence and outcome of AMI and its subtypes, and the applied diagnostics and treatments of different forms of AMI. For comparison of demographic, clinical and laboratory variables between two groups, Fisher’s exact test or Mann–Whitney *U* test were used as appropriate. Statistical significance was defined as *p* < 0.05. SPSS and *R* statistical packages were used for analyses. Complete-case analysis was used in case of missing data.

Incidence of AMI among adult patients hospitalized in acute care facilities was calculated as follows for each site: number of patients with confirmed AMI/total number of adult admissions in the hospital (excluding chronic/long-term care and psychiatry) during the study period. Subgroup analysis in confirmed vs. suspected ischaemia was performed for demographic, clinical and laboratory data at baseline, and for in-hospital mortality. Subgroup analyses based on different subtypes of mesenteric ischaemia (arterial occlusion, venous occlusion, non-occlusive mesenteric ischaemia and other/unclear mechanism) were performed for baseline characteristics, management and outcome. Subgroup analysis of non-delayed vs. delayed diagnosis of AMI based on subjective evaluation of investigators documenting “no delay” or specific factors contributing to delay was performed to identify factors associated with delay in diagnosis.

## Results

### Epidemiology

**Fig. 1:**
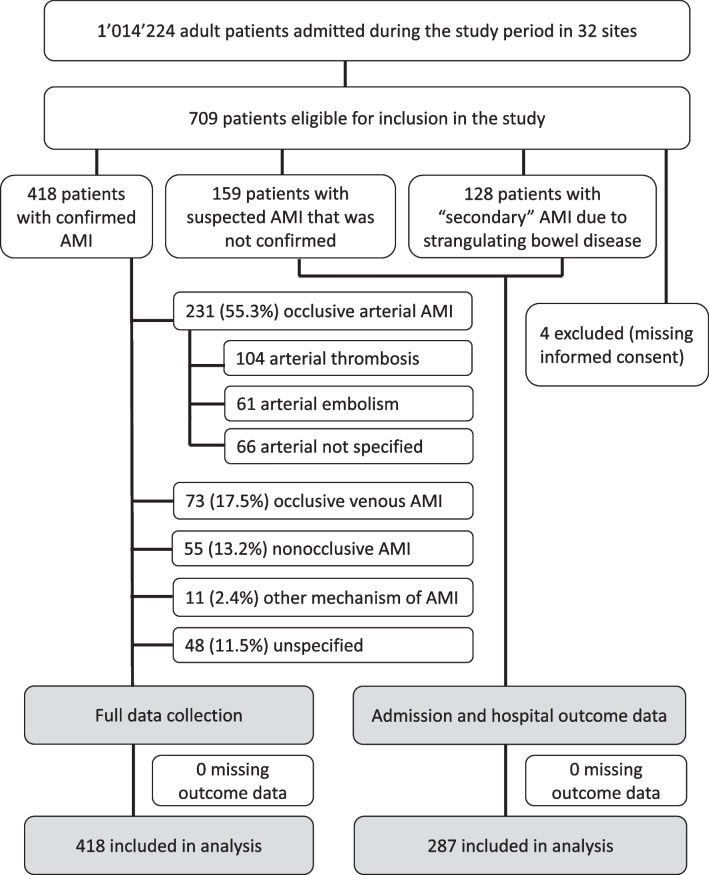
Flow chart. Legend: AMI—acute mesenteric ischaemia

**Fig. 2:**
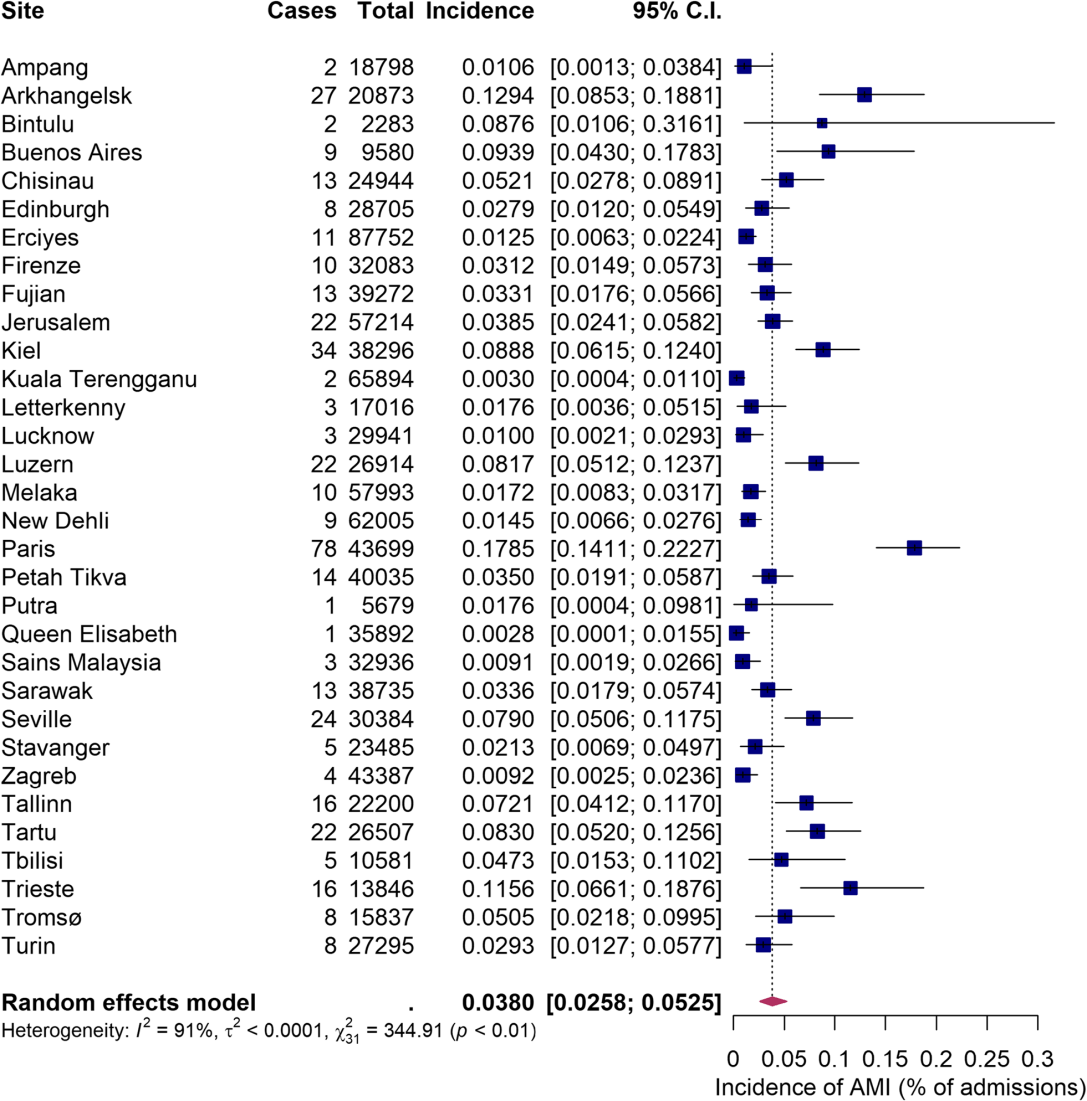
Incidence of AMI (% of admissions) in different sites and in total

In total, 705 patients from 32 sites (17 from European, 14 from Asian and 1 from South American continent) with 31 145 acute care beds were recruited in the study (Additional file 1: Table S2). This number included 418 patients with confirmed AMI, 159 patients (from 25 sites) with suspected but ultimately not confirmed AMI and 128 patients (from 25 sites) with local intestinal gangrene due to SBO. Four eligible patients were excluded due to a consent form being missing. The study flow chart is presented in Fig. 1. Overall proportion of AMI was 0.038% (95%CI 0.0258–0.0525) of adult patients hospitalized in acute care hospitals, with a wide variability between the sites (Fig. 2). Overall proportion among adult patients admitted to acute care hospitals was 0.018% (95%CI 0.012–0.025) for arterial occlusive AMI, 0.004% (0.002–0.007) for venous occlusive AMI and 0.0012 (0.0004–0.0037) for NOMI. The number of sites reporting different types of AMI is presented in Table 1.

**Table 1:** Proportions and mortality of the different forms of acute mesenteric ischaemia

AMI type (n of sites)^a^	Number of cases	Hospital mortality	90 days mortality
Arterial occlusive (*n* = 30)	231 (55.3%)	114 (49.4%)	129 (55.8%)
Arterial embolism (*n* = 21)	61 (14.6%)	33 (54.1%)	34 (55.7%)
Arterial thrombosis (*n* = 28)	104 (24.9%)	47 (45.2%)	57 (54.8%)
Arterial unspecified (*n* = 18)	66 (15.7%)	34 (51.5%)	38 (57.6%)
Venous thrombosis (*n* = 19)	73 (17.5%)	10 (13.7%)	12 (16.4%)
NOMI (*n* = 13)	55 (13.2%)	40 (72.7%)	41 (74.5%)
Other (*n* = 7)	11 (2.6%)	7 (63.6%)	7 (63.6%)
Unclear (*n* = 18)	48 (11.5%)	34 (70.8%)	34 (70.8%)
TOTAL (*n* = 32)	418 (100%)	205 (49.0%)	223 (53.3%)

Other forms include traumatic or non-traumatic dissection, mechanical causes due to tumour or bowel distortion, AMI after angiographic embolization of branches due to bleeding, mechanical devices for cardiac support and abdominal compartment syndrome

(n)^a^ indicates the number of sites where at least one case of this type of AMI was reported

*NOMI* non-occlusive mesenteric ischaemia

#### Confirmed versus suspected AMI

**Fig. 3:**
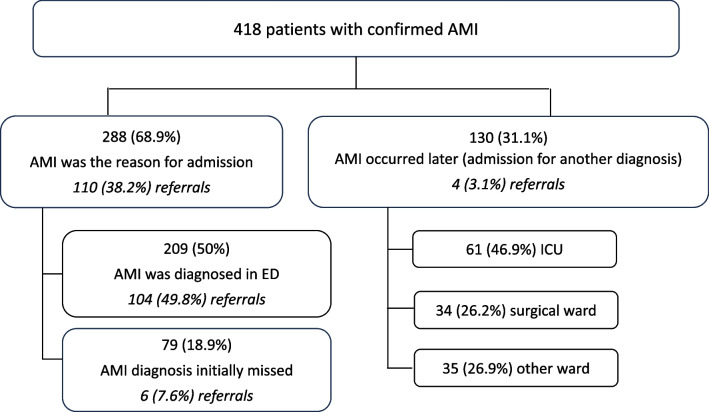
Locations and timing of diagnosis. Legend: *AMI*—acute mesenteric ischaemia; *ED*—emergency department; *ICU*—intensive care unit

Diagnosis of AMI was most frequently confirmed made by CT-scan 303/418 (72.5%) (Additional file 1: Table S3) and at surgery 188/418 (45.0%), and less frequently by histology 34/418 (8.1%), angiography 22/418 (5.3%) and endoscopy 15/418 (3.6%). In total, five cases were confirmed at autopsy (1.2%), and four of those had not been identified previously by another method. AMI was the primary reason for hospital admission in 288/418 (68.9%) of patients with confirmed AMI, while 130/418 (31.1%) developed AMI during their hospital stay after admission for a different primary diagnosis (Fig. 3), at a median day 3 (1–12) after admission. However, in the secondary diagnosis group, 50/130 (38.5%) patients had abdominal pathology (including intra-abdominal infection/suspected peritonitis, gastrointestinal haemorrhage etc.) recorded as their primary diagnosis, compatible with the possibility that AMI was initially missed.

Of note, more than half of patients admitted via the emergency department (ED) and with confirmed AMI, travelled to hospitals by their own means (i.e. were not brought by ambulance) (Additional file 1: Table S4). One site, an intestinal stroke unit, had the largest absolute number as well as proportion of confirmed AMI included exclusively patients with confirmed AMI (no patients with suspected AMI), and 77/78 (98.7%) of cases at that site were referred from another hospital (composing 67.5% of the total number of 114 referrals). Of those 78 patients at that site, AMI was caused by arterial occlusion in 52 (66.7%), venous occlusion in 25 (32.1%), other mechanism in 1 (1.3%) patient (and NOMI in zero patients). In a sensitivity analysis, this selection bias due to being a tertiary referral unit influenced comparisons between confirmed, suspected and SBO patients. Therefore, this particular site was excluded from the final analysis for comparison of suspected vs. confirmed AMI (Table 2), although data from this site were included in other analyses.

**Table 2:** Baseline characteristics and hospital mortality of patients with confirmed AMI compared to those with suspected but not confirmed AMI

	No AMI *N* = 159	SBO *N* = 128	AMI *N* = 340	*P* No AMI vs. AMI	*P* SBO vs. AMI
*Demographics*					
Gender (male), *n* (%) (*n* = 627)	88 (55.3)	60 (46.9)	198 (58.2)	0.561	0.004
Age, median (range) (*n* = 627)	69 (23–97)	70.5 (24–96)	71 (18–99)	0.331	0.873
BMI (*n* = 465)	24.9 (22.9–27.7)	24.2 (20.4–27.7)	25.1 (22–28.3)	0.836	0.114
*Previous health*					
Disability^a^, *n* (%) (*n* = 590)	43 (27.0)	32 (25.0)	75 (22.1)	0.257	0.020
Smoking, *n* (%) (*n* = 469)					
Current	31 (19.5)	20 (15.6)	67 (19.7)	0.543	0.312
Former	26 (16.4)	15 (11.7)	40 (11.8)		
Previous AF, *n* (%) (*n* = 604)	34 (21.4)	24 (18.8)	93 (27.4)	0.263	0.055
Previous myocardial infarction, *n* (%) (*n* = 600)	23 (14.5)	10 (7.8)	77 (22.6)	0.040	< 0.001
Thromboembolism, *n* (%) (*n* = 590)	17 (10.7)	7 (5.5)	60 (17.6)	0.045	0.001
Arterial	9 (52.9)	3 (42.9)	37 (61.7)		
Venous	8 (47.1)	4 (57.1)	22 (36.7)	0.576	0.420
Charlson comorbidity index (*n* = 582)	4 (2–6)	4 (2–5)	4 (2–6)	0.401	0.022
*Acute condition*					
APACHE II, points (*n* = 371)	16 (8–22)	10 (6–14)	17 (11–24)	0.063	< 0.001
SOFA, points (*n* = 374)	3 (1–8)	2 (0–4)	5 (2–10)	0.005	< 0.001
New AF, *n* (%) (*n* = 627)	6 (3.8)	4 (3.1)	33 (9.7)	0.020	0.020
Mechanical ventilation, *n* (%) (*n* = 627)	46 (28.9)	45 (35.2)	154 (45.3)	< 0.001	0.059
Vasopressors, *n* (%) (*n* = 627)	30 (18.9)	9 (7.0)	104 (30.6)	0.007	< 0.001
*Symptoms suggesting AMI*					
Acute abdominal pain, *n* (%)	126 (79.2)	120 (93.8)	260 (76.5)	0.566	< 0.001
Diarrhoea, *n* (%)	23 (14.5)	7 (5.5)	47 (13.8)	0.890	0.014
Bloody stool, *n* (%)	13 (8.2)	5 (3.9)	35 (10.3)	0.517	0.026
Shock, *n* (%)	29 (18.2)	5 (3.9)	90 (26.5)	0.055	< 0.001
WBC, cells × 10^9^ (*n* = 604)	12.7 (8–18.2)	12.4 (8.2–16)	16.2 (11.5–21.0)	< 0.001	< 0.001
CRP, mg/L (*n* = 471)	45 (10–123)	36 (6–112)	108 (31–225)	< 0.001	< 0.001
Creatinine, µmol/L (*n* = 592)	101 (75–146)	90 (71–138)	127 (88–208)	< 0.001	0.010
eGFR, ml/min/1.73 m^2^ (*n* = 426)	59 (32–85)	60 (40–84)	44 (21–68)	0.005	< 0.001
ASAT, U/L (*n* = 525)	32 (21–118)	24 (18–32)	46 (26–112)	0.075	< 0.001
Troponin T, ng/L (*n* = 179)	49 (11–240)	30 (10–100)	53 (16–809)	0.538	0.969
pH (*n* = 478)	7.34(7.21–7.40)	7.38 (7.34–7.42)	7.32 (7.23–7.4)	0.919	0.124
BE (*n* = 459)	− 4.1 (− 11.5; 0.2)	− 0.8 (− 5.5; 2)	− 5.5 (− 11; − 0.5)	0.585	< 0.001
D-dimers, mg/L (*n* = 120)	6 (4–16)	1.45 (0.34–5)	7 (2.3–17)	0.997	< 0.001
Lactate, mmol/L (*n* = 487)	2.95 (1.55–7.1)	2.15 (1.5–3.4)	4 (2.0–7.1)	0.052	< 0.001
*Outcome*					
Hospital mortality *n* (%) (*n* = 627)	42 (26.4)	19 (14.8)	198 (58.4)	< 0.001	< 0.001

This analysis includes patients in whom AMI was suspected at any time point (hospital admission or later). Excluded are patients from one site with no suspected patients and 77/78 referred from another hospital. SBO refers to patients with local intestinal ischaemia due to strangulating bowel disease

*AF* atrial fibrillation, *AMI* acute mesenteric ischaemia, *APACHE* Acute Physiology and Chronic Health Evaluation, *ASAT* aspartate aminotransferase, *BE* base excess, *BMI* body mass index, *CRP* C-reactive protein, *eGFR* estimated glomerular filtration rate, *SOFA* sequential organ failure assessment, *WBC* white blood cell count

^a^Disability was defined as need for any assistance in everyday life

(*n* = *x*) after the name of the variable always indicates number of patients included in the analysis for this specific variable. Data are presented in median (interquartile range) if not stated otherwise

Full data including all sites are provided in Additional file 1: Table S4. Sensitivity analysis to explore the impact of “referral” on the baseline values was performed, identifying a similar pattern regarding suspected vs. confirmed AMI analysis. With the exception of the tertiary referral unit that had different patient characteristics, there was no difference in demographics and clinical characteristics of patients presenting directly via the ED compared to those referred in from other sites (*n* = 37) (Additional file 1: Table S5) and they were kept in the analysis comparing suspected vs. confirmed AMI.

Patients with confirmed AMI more often had a history of myocardial infarction and thromboembolic events and were more severely ill compared to those with suspected but eventually not confirmed AMI (Table 2). Compared to patients with local intestinal gangrene due to SBO, patients with confirmed AMI had less previously recorded disability (need for assistance), were more often male and had history of myocardial infarction (19%) and thromboembolic events (14%). Those with confirmed AMI were more severely ill with more abnormal laboratory values on admission, less often reported abdominal pain as their main symptom, and more often suffered diarrhoea. Lactate values within 0–12 h before the diagnosis were higher in patients with confirmed AMI compared to patients with SBO (p < 0.001) and those with suspected but not confirmed AMI (*p* = 0.052) (Table 2). Patients with confirmed AMI had higher hospital mortality [198/340 (58.4%)] with one site excluded than patients with suspected but not confirmed AMI [42/159 (26.4%)], and patients with SBO [19/128 (14.8%)], all *p* < 0.001.

#### Subtypes of AMI

The proportions of different subtypes of AMI and the associated mortality are presented in Table 1. In a subset of patients 48/418 (11.5%), the aetiological mechanism remained unclear. Comparisons of baseline characteristics and outcome of different types of AMI are presented in Table 3. Occlusive AMI was most often diagnosed in ED, with 46/231 (19.9%) of arterial occlusive and 11/73 (15.1%) of venous thrombosis being diagnosed later during the hospital stay. The majority of cases of NOMI occurred during the hospital stay due to another distinctive diagnosis 40/55 (72.7%), and most of those patients were being treated in a ICU setting 35/55 (63.6%). Compared to other subtypes of AMI, patients with venous AMI were younger, previously healthier (but with a significantly higher rate of previous venous thromboembolic events) and less severely ill at admission despite similar elevation of inflammatory markers and more elevated D-dimer levels (Table 3). This group had better outcome than other types of AMI. Patients with NOMI were the most severely ill and had higher lactate values during 24–48 h before diagnosis, whereas levels of inflammatory markers were similar to other subtypes of AMI.

**Table 3:** Baseline characteristics and outcome in patients with different subtypes of AMI

AMI subtype	All subtypes *N* = 418	Arterial occlusive *N* = 231 (55.3%)	Venous *N* = 73 (17.5%)	NOMI *N* = 55 (13.2%)	Other/Unclear^a^ *N* = 59 (14.1%)	*P* Art versus Ven	*P* Art versus NOMI
*Variable*							
Demographics							
Gender (male), *n* (%) (*n* = 418)	241 (57.7)	131 (56.7)	45 (61.6)	34 (61.8)	31 (52.5)	0.498	0.545
Age, median (range) (*n* = 418)	70 (18–99)	71 (23–99)	64 (18–94)	70 (18–93)	72 (21–94)	< 0.001	0.649
BMI, kg/m^2^ (*n* = 312)	24.9 (21.8–28.2)	24.3 (21.3–27.4)	25.9 (23.3–31.2)	26.1 (23.5–28.3)	23.6 (20–27.7)	0.004	0.080
Previous health/medication							
Disability^b^, *n* (%) (*n* = 392)	83 (19.9)	49 (21.2)	5 (6.8)	16 (29.1)	13 (22.0	0.004	0.210
AF, *n* (%) (*n* = 408)	102 (24.4)	73 (31.6)	6 (8.2)	13 (23.6)	10 (16.9)	< 0.001	0.254
AH, *n* (%) (*n* = 409)	269 (64.4)	166 (71.9)	27 (37.0)	37 (67.3)	39 (66.1)	< 0.001	0.314
Previous MI, *n* (%) (*n* = 403)	80 (19.1)	48 (20.8)	3 (4.1)	20 (36.4)	9 (15.3)	< 0.001	0.035
Thromboembolism, *n* (%) (*n* = 395)	62 (14.8)	30 (13.0)	15 (20.5)	11 (20.0)	6 (10.2)	0.187	0.285
Arterial	39 (63.9)	25 (83.3)	2 (13.3)	7 (63.6)	5 (83.3)		
Venous	22 (36.1)	4 (13.3)	13 (86.7)	4 (36.4)	1 (16.7)	< 0.001	0.182
Charlson comorbidity index (*n* = 383)	4 (2–6)	4 (3–6)	2 (1–4)	5 (3–6)	4 (2–6)	< 0.001	0.508
Anticoagulants, *n* (%) (*n* = 392)	110 (26.3)	70 (30.3)	20 (27.4)	9 (16.4)	11 (18.6)	0.558	0.029
Antiplatelets, *n* (%) (*n* = 386)	123 (29.4)	77 (33.3)	10 (13.7)	27 (49.1)	9 (15.3)	< 0.001	0.060
Acute conditions at baseline							
APACHE II, points (*n* = 418)	15 (9–23)	15 (10–21)	8 (4–12)	25 (18–30)	17 (8–24)	< 0.001	< 0.001
SOFA, points (*n* = 418)	4 (2–9)	3 (1–7)	2 (1–3)	11 (9–14)	5.5 (2–9)	0.010	< 0.001
New AF, *n* (%) (*n* = 418)	33 (7.9)	23 (10.0)	0	8 (14.5)	2 (3.4)	0.002	0.337
MV, *n* (%) (*n* = 418)	166 (39.7)	89 (38.4)	9 (12.3)	37 (67.3)	31 (52.5)	< 0.001	< 0.001
Vasopressors, *n* (%) (*n* = 418)	112 (26.8)	52 (22.5)	5 (6.8)	36 (65.5)	19 (32.2)	0.002	< 0.001
Laboratory results							
WBC, cells × 10^9^ (*n* = 404)	16 (11.1–21)	16.2 (11.4–20.3)	14.7 (10.6–22)	16.0 (10–21)	17 (12.7–21.8)	0.311	0.628
CRP, mg/L (*n* = 339)	100 (30–213)	95 (21–215)	106 (40–166)	108 (39–258)	139 (66–274)	0.668	0.239
Creatinine, µmol/L (*n* = 393)	113 (78–190)	112 (76–179)	83 (66–107)	194 (121–311)	139 (95–218)	< 0.001	< 0.001
eGFR, ml/min/1.73m^2^ (*n* = 294)	55 (27–94)	50 (26–80)	82 (54–103)	26 (10–45)	43 (21–60)	< 0.001	< 0.001
ASAT, U/L (*n* = 338)	39 (24–82)	39 (23–72)	28 (20–35)	122 (43–408)	47 (28–135)	< 0.001	< 0.001
Amylase, U/L (*n* = 198)	63 (35–132)	66 (41–152)	45 (28–54)	114 (50–147)	64 (30–151)	0.002	0.204
Troponin T, ng/L (*n* = 160)	40 (13–134)	31 (13–124)	12 (12–20)	141 (60–1071)	48 (10–89)	0.007	< 0.001
pH (*n* = 322)	7.33 (7.23–7.4)	7.33 (7.23–7.4)	7.39 (7.3–7.44)	7.26 (7.2–7.36)	7.3 (7.2–7.4)	0.017	0.045
BE (*n* = 256)	− 6 (− 11; -1)	− 5 (− 11; 0)	− 1.5 (− 4, 1.)	− 8 (− 14, -5)	− 7 (− 12–3)	0.017	0.020
D-dimers, mg/L (*n* = 119)	5 (2–10)	4 (1.25–10)	8 (5–13)	5.5 (2–7)	6 (0.65–17)	0.028	0.550
Lactate, mmol/L (*n* = 349)	3.1 (1.6–6.7)	3.2 (1.7–6.9)	1.6 (1.3–2.6)	4.3 (2–8.5)	4.2 (2–7.3)	< 0.001	0.065
Outcomes							
Hospital mortality *n* (%) (*n* = 418)	205 (49.0)	114 (49.4)	10 (13.7)	40 (72.7)	41 (69.5)	< 0.001	0.002
Discharged *n* (%)^c^, (*n* = 418) Home	146 (34.9/68.5)	78 (33.8/66.7)	55 (75.3/87.3)	4 (7.3/26.7)	9 (15.3/50)		
Health-care facility	67 (16.0/31.5)	39 (16.9/33.3)	8 (11.0/12.7)	11 (20.0/73.3)	9 (15.3/50)	0.024	< 0.001
Hospital LOS, days(*n* = 415)	11 (3–20)	9 (3–18)	12 (7–19)	13 (3–37)	13 (2–23)	0.020	0.096
ICU stay, days (*n* = 280)	5 (2–13)	4 (2–10)	5 (2–10)	6 (2–27)	4.5 (1–12)	0.723	0.109
MV duration, days (*n* = 218)	3 (1–10)	3 (1–8)	3 (1–6)	10 (2–20)	2 (0.4–6)	0.746	0.003
RRT *n* (%) (*n* = 418)	81 (19.4)	39 (16.9)	5 (6.8)	29 (52.7)	8 (13.6)	0.036	< 0.001
PN, days (*n* = 131)	10 (4–18)	9 (4–16)	12 (4–19)	14 (4–20)	16 (4–71)	0.479	0.249
Stoma at discharge, *n* (%) (*n* = 213)	58 (13.9)	32 (13.9)	10 (13.7)	4 (7.3)	12 (20.3)	0.098	1
PN at discharge, *n* (%) (*n* = 213)	31 (7.4)	17 (7.4)	7 (9.6)	1 (1.8)	6 (10.2)	0.648	0.692
30-day mortality, *n* (%) (*n* = 418)	198 (47.4)	113 (48.9)	9 (12.3)	36 (65.5)	40 (67.8)	< 0.001	0.035
90-day mortality, *n* (%) (*n* = 418)	223 (53.3)	129 (55.8)	12 (16.4)	41 (74.5)	41 (69.5)	< 0.001	0.014

(*n* = x) after the name of the variable always indicates number of patients included in the analysis for this specific variable. Data are presented in median (interquartile range) if not stated otherwise

*AF* atrial fibrillation, *AH* arterial hypertension, *Art* arterial occlusive AMI, *ASAT* aspartate aminotransferase, *BE* base excess, *CRP* C-reactive protein, *eGFR* estimated glomerular filtration rate, *ICU* intensive care unit, *LOS* length of stay, *MI* myocardial infarction, *MV* mechanical ventilation, *NOMI* non-occlusive mesenteric ischaemia, *PN* parenteral nutrition, *RRT* renal replacement therapy, *SOFA* sequential organ failure assessment, *Ven* venous AMI, *WBC* white blood cells

^a^Other (*n* = 11; 2.6%): included specific mechanisms such as dissection, bowel distortion, mechanical devices for cardiac support and abdominal compartment syndrome/Unclear (*n* = 48; 11.5%)

^b^Disability was defined as need for any assistance in everyday life

^c^in parenthesis: percentage of all patients/percentage of discharged patients

### Diagnosis of AMI

The following symptoms supported the suspicion of AMI (occurring at any time point): acute abdominal pain in 336/418 (80.4%); shock in 90/418 (21.5%), diarrhoea in 73/418 (17.5%) and bloody stool in 39/418 (9.3%) patients (Additional file 1: Table S4). Other symptoms supporting the suspicion of AMI were reported in 72/418 patients (17.2%) and included nausea/vomiting in 42/418 (10.0%); abdominal distension in 12/418 (2.9%), absence of passage in 6/418 (1.4%) and other factors/conditions such as hyperlactatemia, systemic infection/sepsis or intra-abdominal hypertension in 15/418 (3.6%). In 7/418 (1.7%) patients with confirmed AMI, no symptoms suggestive for AMI were reported.

Most commonly-performed laboratory tests in patients with confirmed AMI were WBC, creatinine, lactate, CRP and ASAT (all measured in > 80% of patients) (values presented in Table 2 and Additional file 1: Table S4).

Computerized tomography (CT) scan was used as the primary radiological study in 300/418 (71.8%), followed by plain x-ray in 65/418 (15.6%) and ultrasound in 35/418 (8.4%) of patients with eventually confirmed AMI. Ultimately, CT-scan was performed in 369/418 (88.3%) patients with AMI, in 158/418 (42.8%) CT-scan with both arterial and venous or late phase enhancement was performed, in 91/418 (24.7%) only arterial, in 62/418 (16.8%) only venous or delayed phase and in 58/418 (15.7%) without contrast. Contrast enhancement protocols during CT in different subtypes of AMI are presented in Additional file 1: Table S3.

A radiologist diagnosed AMI in 298/418 (71.3%) of cases, with median response time of 30 (15–60) min. The suspicion of AMI was mentioned in the referral to the radiologist in 197/418 (47.1%) patients with confirmed AMI. In total, 51 cases of AMI were diagnosed at surgery without a previous suspicion of AMI.

No delay in diagnosis was reported by investigators in 78 (27.1%), delay in 75 (26.0%) of patients with eventually confirmed AMI admitted via ED with symptoms of AMI (288 out of 418 included AMI patients). In 135/288 (46.9%) patients, the investigators were not decisive regarding delay vs. no delay and these patients were excluded from this particular analysis.

Time in the hospital until diagnosis in “delayed” cases was 12 (6–12) versus 3 (2–6) hours in patients without “delay” (*p* < 0.001) and time to treatment 8 (6–20) versus 4 (2–8) hours (*p* < 0.001), respectively. However, time elapsed from the beginning of symptoms until presentation to the hospital was in general very long: 24 (8–72) in “no delay” versus 20 (5–48) hours in “delayed”, *p* = 0.244 (Additional file 1: Table S6).

Factors associated with “no delay” were suspicion of AMI mentioned in the referral letter for the first radiological study; and “radiologist diagnosed AMI”. However, hospital mortality was not significantly different between “no delay” and “delayed” groups 42/78 (53.8%) versus 36/75 (48.0%), respectively, *p* = 0.519).

### Management of AMI

Open surgery was most often used as the primary treatment [242/418 (57.8%)] (Table 4). In the majority of cases, this was gastrointestinal surgery only, with bowel resection performed in 134/418 (31.1%). Surgical revascularization was performed in 34/418 (8.1%) patients (in 14 cases concomitantly with bowel resection), and endovascular revascularization was combined with surgery in 25/418 (6.0%). Endovascular revascularization alone was applied in 54/418 (12.9%) and conservative—only pharmacological and/or supportive—treatment in 76/418 (18.2%). Palliative care without any attempt of treatment with curative intention was applied in 46/418 (11.0%). Additionally, end-of-life care was initiated secondarily after initial curative attempt in 88 patients (23.6% of 373 initially treated with a curative attempt).

**Table 4:** Initial management of AMI and its different subtypes

	All *n* = 418	Arterial *n* = 231	Venous *n* = 73	NOMI *n* = 55	Other^a^ *n* = 11	Unclear *n* = 48
*Surgical only,* *n* (%)	217 (52.0)	113 (48.7)	31 (42.5)	28 (51.9)	7 (63.6)	38 (79.2)
Laparoscopy	17 (4.1)	6 (2.6)	4 (5.5)	1 (1.8)	1 (9.1)	5 (10.4)
Explorative laparoscopy	10 (2.4)	5 (2.2)	1 (1.4)	1 (1.8)	–	3 (6.3)
Laparoscopic bowel resection	7 (1.7)	1 (0.4)	3 (4.1)	–	1 (9.1)	2 (4.2)
Laparotomy	200 (47.8)	107 (46.3)	27 (37)		6 (54.5)	33 (68.8)
Explorative laparotomy	39 (9.3)	19 (8.2)	4 (5.5)	7 (12.7)	–	9 (18.8)
Surgical revascularization	20 (4.8)	19 (8.2)	1 (1.4)	–	–	–
Revascularization and bowel resection	14 (3.3)	12 (5.2)	1 (1.4)	–	1 (9.1)	–
Bowel resection	127 (30.4)	57 (24.7)	21 (28.8)	20 (36.4)	5 (45.5)	24 (50)
*Surgical and endovascular,* *n*(%)	25 (6.0)	22 (9.5)	–	1 (1.8)	2 (18.2)	–
Endovascular revascularization with explorative laparotomy	1 (0.2)	1(0.43)				–
Endovascular revascularization with bowel resection	7 (1.7)	6 (26)			1 (9.1)	–
Hybrid revascularization with explorative laparotomy	6 (1.4)	6 (26)				–
Hybrid revascularization with bowel resection	11 (2.6)	9 (3.9)		1 (1.8)	1 (9.1)	–
*Endovascular,* *n* (%)	54 (12.9)	51 (22.0)	2 (2.7)	–		1 (12.1)
Aspiration of thrombus/embolus	18 (4.3)	17 (7.3)	1 (1.4)	–		–
Balloon dilatation	10 (2.4)	8 (3.4)	1 (1.4)	–		1 (2.1)
Stenting	25 (6.0)	25 (10.8)	–	–		–
Thrombolysis	11 (2.6)	11 (4.7)	–	–		–
Combined	1 (0.2)	–	1 (1.4)	–		–
*Interventions in total*						
Revascularization in total, *n* (%)	113 (27.0)	104 (45.0)	4 (5.5)	1 (1.8)	3 (27.3)	1 (2.1)
Bowel resection without revascularization, *n* (%)	134 (32.1)	58 (25.1)	24 (32.9)	20 (36.4)	6 (54.5)	26 (54.2)
Bowel resection in total, *n* (%)	166 (39.7)	85 (36.8)	25 (34.2)	21 (38.2)	9 (81.8)	26 (54.2)
Small bowel resection, *n* (%)	126 (30.1)	71 (30.7)	23 (31.5)	13 (23.6)	7 (63.6)	12 (25)
Residual small bowel length < 200 cm, *n* (%)	44 (10.5)	31 (13.4)	3 (4.1)	4 (7.3)	4 (36.4)	2 (4.2)
Large bowel resection, *n* (%)	84 (20.1)	40 (17.3)	2 (2.7)	17 (30.9)	6 (54.5)	19 (39.6)
Open abdomen, *n* (%)	90 (21.5)	52 (22.5)	11 (15.1)	14 (25.5)	6 (54.5)	7 (14.6)
*Conservative only,* *n* (%)	76 (18.2)	19 (8.2)	39 (53.4)	18 (33.7)	–	–
Full anticoagulation	65 (15.6)	16 (6.9)	38 (52.1)	11 (20.4)	–	–
Prophylactic anticoagulation	4 (1)	1 (0.4)	1 (1.4)	2 (3.7)	–	–
Antiplatelet therapy	17 (4.1)	8 (3.4)	1 (1.4)	8 (14.5)	–	–
*End-of-life care,* *n* (%)	46 (11.0)	26 (11.2)	1 (1.4)	8 (14.5)	2 (18.2)	9 (18.8)

*NOMI* non-occlusive mesenteric ischaemia

^a^Other included specific mechanisms such as dissection, bowel distortion, mechanical devices for cardiac support and abdominal compartment syndrome

Overall, revascularization was undertaken in 113/418 patients (27.0%) patients (Table 4). Among patients with arterial occlusive AMI (*n* = 231), the overall revascularization rate was 45% (endovascular in 55.8%, open surgical in 29.8%, and hybrid in 14.4%). From all revascularizations in arterial occlusive AMI, 40/104 (38.5%) were performed in the largest site with selected patients.

The initial management of AMI is presented in Table 4, and the secondary management is presented in Table 5. Systemic management is summarized in Additional file 1: Table S7.

**Table 5:** Secondary management after initial treatment with curative intention

	All *n* = 372^a^	Arterial *n* = 205^a^	Venous *n* = 72^a^	NOMI *n* = 47^a^	Other^b^ *n* = 9^a^	Unclear *n* = 39^a^
No secondary intervention, *n* (%)	218 (58.7)	114 (55.6)	63 (87.5)	22 (46.8)	1 (11.1)	18 (46.2)
Second look planned	127 (34.1)	83 (40.5)	11 (15.3)	12 (25.5)	5 (55.6)	16 (41)
Preplanned second look performed	93 (24.9)	56 (27.3)	8 (11.1)	12 (25.5)	5 (55.6)	12 (30.8)
Resulted in secondary bowel resection	43 (11.5)	26 (12.7)	3 (4.2)	5 (10.6)	4 (44.4)	5 (12.8)
Bowel resection, *n* (%)	66 (17.7)	41 (20)	6 (8.3)	7 (15.2)	6 (66.7)	6 (15.4)
After initial endovascular treatment *n* = 54	12 (3.2)	12 (5.9)	–	–	–	–
After surgical and endovascular treatment *n* = 25	10 (2.7)	7 (3.4)	–	1 (2.1)	2 (22.2)	–
After initial surgery with revascularization *n* = 34	9 (2.4)	8 (3.9)	–	–	1 (11.1)	–
After initial bowel resection *n* = 134	29 (7.8)	10 (4.9)	5 (6.9)	5 (10.6)	3 (33.3)	6 (15.4)
After explorative laparoscopy/laparotomy *n* = 49	2 (0.5)	2 (1)	–	–	–	–
After initial conservative treatment *n* = 76	6 (1.6)	4 (2.0)	1 (1.4)	1 (2.1)	–	–
End-of-life care, *n* (%)	88 (23.6)	50 (24.4)	3 (4.2)	18 (39.1)	2 (22.2)	15 (38.5)

*NOMI* non-occlusive mesenteric ischaemia

^a^Only patients with initial treatment with curative intention (any method) were included in this analysis, patients in whom end-of-life care was initiated without an attempt of any treatment with curative intention, were excluded

^b^Other included specific mechanisms such as dissection, bowel distortion, mechanical devices for cardiac support and abdominal compartment syndrome

Second-look surgery was pre-planned in 127/242 (52.5%) of patients after the initial surgery, actually performed in 93 (38.4%) and resulted in additional bowel resection in 43 (17.8%). In 10 patients with pre-planned second look, treatment goal was changed to palliation and 6 patients died within the next 2 days, for the remaining 18 patients the reason to deviate from the initial plan was not documented.

All treatment options were available in 309/418 (73.9%) of cases, and intra-arterial vasodilation was not available in 87/418 (20.8%), endovascular treatment in 71/418 (17.0%) and surgery in 13/418 (3.1%) of cases. The most commonly discussed treatment options by teams were exploratory laparotomy 229/418 (54.8%), intestinal resection without revascularization 148/418 (35.4%), endovascular revascularization alone or followed by surgery 139/418 (33.2%), surgical revascularization with or without bowel resection 131/418 (31.3%) and palliation 104/418 (24.9%).

Treatment options were not discussed with patient and/or family in 80/418 cases (19.1%). Different treatment options were discussed in remaining 338/418 (80.9%) cases, including open surgery in 202/418 (48.3%), exploratory laparotomy in 189/418 (45.2%), palliation in 93/418 (22.2%) and endovascular approach (alone or combined with surgery) in 83/418 (19.9%). A comparison of patients offered any active treatment vs. initiating end-of-life care without an attempt at curative treatment is provided in Additional file 1: Table S8.

### Outcome of AMI

Hospital outcomes and 90-day mortality in total and in different types of AMI are presented in Table 3. Overall hospital mortality of patients with AMI was 205/418 (48.8%), being the lowest in venous AMI 10/73 (13.7%) and the highest in NOMI 40/55 (72.7%).

The majority of hospital survivors with venous AMI, 55/63 (87.3%), were discharged home, compared to only 4/15 (26.7%) of survivors with NOMI.

Hospital mortality in the specialized intestinal stroke unit, treating selected, tertiarily referred patients with active endovascular revascularization strategy, was 6/78 (7.7%) and 90 days mortality 13/78 (16.7%). In total, 277/418 (66.3%) patients with AMI were treated in the ICU for median duration of 5 (2–13) days, 166/418 (39.7%) were mechanically ventilated for a median of 3 (1–10) days and 81/418 (19.4%) received renal replacement therapy during their hospital stay. Fifty-eight (13.9% of all, 27.2% of survivors) patients had stoma, and 31 (7.4% of all, 14.6% of survivors) were on parenteral nutrition at hospital discharge. Overall, 146 patients (34.9% of total, 68.5% of survivors) were discharged to home.

## Discussion

In this large multicentre international prospective study, we report the incidence, patient characteristics, diagnostic pathways, management modalities and outcomes of AMI and its different subtypes.

### Incidence of AMI and comparison of suspected versus confirmed AMI

The overall occurrence rate of confirmed AMI was lower than anticipated based on previous analyses [1, 2]. There may be several explanations for this finding, but the most likely is variability in case ascertainment, i.e. that not all patients with AMI were identified in all sites. The highly variable rates of confirmed AMI, suspected but not confirmed AMI, and NOMI, support this hypothesis. The reason for below-expected case ascertainment may reflect a variable awareness by receiving clinicians, leading to missed diagnosis, but may also reflect the challenge of detecting all patients with symptoms of AMI in different locations within a hospital, and the duration of their hospital stay. Universally low autopsy rates [14, 15] preclude post-mortem diagnosis of AMI in most missed cases.

Specialized or regional centres may have higher incidence rates due to tertiary referrals. While the overall proportion of tertiary referrals was low, one site had almost exclusively patients referred from other hospitals with the diagnosis of AMI already confirmed and already triaged as having potential for revascularization, together resulting in selection bias. That site was the only declared specialized Intestinal Stroke Unit, and perhaps unsurprisingly reported disproportionately better outcomes than might be expected or observed in the generalized denominator of all-comer units. (Additional file 1: Table S4), in keeping with earlier studies [3, 16].

Although that specialist site was excluded from the analysis comparing suspected vs. confirmed AMI, the results from that centre are still valuable when analysing different subgroups. Moreover, the experience of this unit indicates that selected group of patients with occlusive AMI, and a high frequency of revascularization, may be treated with very good results (Additional file 1: Table S4). These data may be helpful in identifying patients where referral is indicated, despite being associated with delay due to interhospital transfer, and in the future, may also help more detailed exploration of factors indicating futility of any treatment attempt. Our results of sensitivity analysis, separating data from the specialized intestinal stroke unit, suggest that case selection (defined as transfer from other institution with potential for active revascularization) is a major determinant of high survival. Even though the effect of active revascularization on survival of unselected patients cannot be directly estimated and is likely smaller, management of occlusive AMI with active revascularization also in patients undergoing bowel resection should be undertaken more frequently in line with current guidelines. Simultaneously, it should be acknowledged that hospitals admitting unselected cases of AMI will be unlikely to be able to achieve results similar to centres with selected patients and active revascularization practices. However, it is plausible that some improvement could be achieved with use of appropriate contrast enhancement of CT-images, confirming the earlier results of Tolonen et al. [17].

We anticipated that this study would recruit more patients with suspected AMI than patients with confirmed AMI, as previously shown in one small study [18]. It is possible that some sites were less motivated to include patients with suspected AMI or that information regarding these patients did not reach investigators before AMI was either confirmed or excluded. Despite this potential for selection bias, patients with suspected but not confirmed AMI were different from patients with confirmed AMI in several metrics, but many similarities indicate that suspicion of AMI was probably raised appropriately, underlining difficulties in diagnosis based on clinical features only.

Patients with localized intestinal gangrene due to SBO were less severely ill compared to patients with AMI. Although this finding was partially expected, it is important because in existing literature SBO (with the extent of gangrene commonly not specified) is often bundled as AMI. In a recent systematic review on biomarkers of AMI, the majority of studies investigating AMI included patients with SBO, complicating interpretation of results [19]. To our knowledge, our study is one of the first that separates these entities and allows for some rough comparisons of data beyond just blood lactate values [20]. However, there was minor overlap between local intestinal gangrene due to SBO and extensive bowel necrosis categorized as “other” subtype of AMI (*n* = 3). Therefore, these results need to be interpreted with caution. Whether patients with SBO and intestinal ischaemia (often transient and/or local) should be considered in the pool of patients with AMI is a legitimate question. From a diagnostic and management point of view, SBO is a different entity with more commonly a clearer clinical presentation and more straightforward management strategy. We hope that this analysis will contribute to achieving a consensus on nomenclature in this regard.

### Different subtypes of AMI

The distribution of different subtypes of AMI was largely similar to literature data [1, 2], but the degree of diagnostic uncertainty was surprising in this prospective study. In more than 10% of cases categorized as uncertain aetiology, and in more than one quarter of patients with arterial occlusive AMI, there was uncertainty regarding the specific mechanism (embolism or thrombosis) underlying the AMI event. It also seems likely that patients with NOMI were missed at several sites, (19/32 sites did not include any patients with NOMI). NOMI is more difficult to diagnose compared to other subtypes of AMI, which probably contributes to an even lower diagnostic awareness. If NOMI is not considered as a possible causative mechanism of multiple organ dysfunction resulting in lethal outcome in an ICU setting, then NOMI may go unrecognized unless autopsy is performed.

### Diagnosis

Previous observations have shown that clinical characteristics and laboratory tests are unable to clearly distinguish between patients with and without AMI [19, 21]. This study revealed differences that can be explored in more detail in the future through an attempt to construct a prediction model. Based on the literature, contrast-enhanced CT-scan has a good accuracy in diagnosing arterial and venous occlusive AMI [21, 22], while being less accurate for diagnosis of NOMI [23]. In our study not always an optimal contrast enhancement was used, however, in a previous study, an optimal CT-protocol was used only in 35% of cases [24].

An important factor in avoiding delay in diagnosis in this study was when a suspicion of AMI was mentioned in referral requests to radiology, as also suggested by previous observations [17, 24]. Correct selection of timing of CT imaging after intravenous contrast enhancement allows appropriate assessment and facilitates diagnosis of AMI by radiologists, and this was also associated with timely diagnosis and treatment in this study. However, a greater delay to intervention seems to occur prior to hospital admission rather than in hospital, with median time of 24 h from beginning of symptoms until arrival to hospital (more than half with self-presentation), confirming the previous results of a retrospective study [2]. This finding stresses the need to improve overall awareness of AMI among the population and first care providers. This time delay factor becomes crucial in patients lacking collateral vessels to maintain mesenteric perfusion, whereas others with sufficient collaterals may be symptomatic for longer time period without developing transmural ischaemia.

### Management

Different management strategies are used for different types of AMI, making it somewhat difficult to report a consensus approach. Additionally, differences between sites in expertise in open vascular surgery and endovascular techniques, as well as patient cohorts that were submitted make comparisons difficult. Interestingly, in most operations, only GI resection without revascularization was performed despite the availability of revascularization in majority of these cases, and in considerable number of cases secondary resection was needed. This may indicate another factor with a potential for improvement through better adherence to current guidelines recommending revascularization before bowel surgery in case of arterial occlusion [9, 10]. The overall revascularization rate of 45% for patients with arterial occlusive AMI was very high compared to a large-scale register study from the USA where only 2.9% received intestinal revascularization [25]. However, more than one-third of these patients came from the Intestinal stroke unit, whereas the pooled overall revascularization rate of occlusive arterial AMI for all the other sites was 36%. A more proactive approach to revascularization may potentially prevent secondary small and large bowel resection in certain cases, which may reduce mortality as well as risk of short bowel syndrome.

### Outcome

The overall mortality of approximately 50% was high, especially considering the prospective nature of the study and the focus on timely diagnosis and treatment. Different subtypes of AMI had different outcomes, which was expected and has also been reported in previous investigations [1, 2]. However, while hospital mortality of arterial occlusive AMI was similar to observations in recent systematic review [1], hospital mortality of venous occlusive AMI was lower (14 vs. 26%) and of NOMI was higher (73 vs 58%). These differences in mortality may be at least partially explained by patient populations from smaller, commonly retrospective single-centre investigations focussing on one specific type of AMI included in the systematic review [1]. The largest site in the current study treated selected patients and provided a large proportion of patients with venous AMI, high proportion of revascularization among those with arterial occlusion and no patients with NOMI, which we interpret as the main causes for the observed differences.

None of the patients in this study were treated with intra-arterial vasodilators, while one earlier study suggested lower mortality of NOMI with this intervention [26]. However, the mortality rate of NOMI in the control group in this study by Takeguchi et al. [26] was two-fold lower compared to our study. Our study showed that patients considered having AMI are very different between different sites, and this cannot entirely be explained by different types of AMI and time elapsed between the onset of symptoms and application of treatment. Therefore, a multicentre approach is important to identify existing differences and move towards more clear and precise definitions in the future.

### Limitations

This study has several limitations. Patients with suspected AMI, but also confirmed AMI, and especially NOMI, were likely missed in several sites leading to a case ascertainment bias. Differences between sites were noticeable in many aspects, including the proportion of patients referred in, differences in CT and contrast enhancement protocols, and subtypes of AMI. The observed study site heterogeneity highlights a intrinsic problem with a multicentre design such as ours when assessing a disease with multifaceted nature in different healthcare systems.

We aimed to document “real world” current clinical practises without influencing investigators by giving them specific guidance on definitions that could be considered as a limitation contributing to heterogeneity. On the other hand, we think that this approach was useful to identify problems with definitions and case ascertainment. While the diagnosis of AMI in case of transmural necrosis at surgery is obvious, current definitions for earlier stages and clinical criteria for suspicion of AMI are probably insufficient and may be improved by a consensus process. We propose using the data and results from this study to initiate such an international consensus process.

There were missing data for several variables, partially explained by uncertainty, e.g. for evaluation “delayed” vs “non-delayed” diagnosis. However, our data allow defining respective time limits for future studies. The prospectively collected data from 32 participation centres in three continents collected into a secure computerized data management system increase generalizability of results to other settings.

## Conclusions

This study identified the occurrence rate of AMI to be 0.038% of adult patients hospitalized in acute care hospitals in different parts of the world, with a diagnosis of AMI resulting in an overall hospital mortality of almost 50%. Low overall revascularization rate and large variations between the sites regarding incidence, baseline characteristics, management and outcome were observed, indicating the need for clearer guidance in diagnosis and management, but also in criteria for suspicion and diagnosis of AMI. Patients frequently arrived at hospital 24 h after onset of symptoms, while the median time in the hospital until diagnosis was 6 h, suggesting potential for improved awareness of AMI in the community and pre-hospital medical services. Early involvement of radiologist is helpful in shortening the time to diagnosis after hospital admission. Active revascularization is seldom undertaken outside specialist centres, despite the potential for improving outcomes, and this is an area where there is opportunity for significant healthcare improvement.

### Supplementary Information

**Additional file 1: **Supplementary Methods and Results.

## Data Availability

Patient level data and the full dataset can be made available from the corresponding author at reasonable request, following data protection rules.

## Abbreviations

AMI: Acute mesenteric ischaemia
ASAT: Aspartate aminotransferase
CRP: C-reactive protein
ICU: Intensive care unit
NOMI: Non-occlusive mesenteric ischaemia
PN: Parenteral nutrition
RRT: Renal replacement therapy
SOFA: Sequential organ failure assessment
WBC: White blood cells
