# Supplementary material for: Incidence, diagnosis, management and outcome of acute mesenteric ischaemia: a prospective, multicentre observational study (AMESI Study)
Source: Crit Care. 2024 Jan 23;28:32. doi: 10.1186/s13054-024-04807-4 (PMC10807222; doi:10.1186/s13054-024-04807-4)
Supplement: Supplementary file 1 — Additional file 1: Supplementary Methods and Results. [file 13054_2024_4807_MOESM1_ESM.docx]

**Additional file 1.**

**Supplementary Methods and Results.**

**Incidence, diagnosis, management and outcome of Acute MESenteric Ischaemia: a prospective, multicentre observational study (AMESI Study)**

**Table S1.** STROBE Statement—Checklist of items that should be included in reports of ***cohort studies***.

|  | Item No | Recommendation | Reported on page No |
| --- | --- | --- | --- |
| **Title and abstract** | 1 | (*a*) Indicate the study’s design with a commonly used term in the title or the abstract | 1 |
|  |  | (*b*) Provide in the abstract an informative and balanced summary of what was done and what was found | 6 |
| Introduction | | |  |
| Background/rationale | 2 | Explain the scientific background and rationale for the investigation being reported | 8 |
| Objectives | 3 | State specific objectives, including any prespecified hypotheses | 9-10 |
| Methods | | |  |
| Study design | 4 | Present key elements of study design early in the paper | 9 |
| Setting | 5 | Describe the setting, locations, and relevant dates, including periods of recruitment, exposure, follow-up, and data collection | 9 |
| Participants | 6 | (*a*) Give the eligibility criteria, and the sources and methods of selection of participants. Describe methods of follow-up | 9-11 |
|  |  | (*b*) For matched studies, give matching criteria and number of exposed and unexposed | NA |
| Variables | 7 | Clearly define all outcomes, exposures, predictors, potential confounders, and effect modifiers. Give diagnostic criteria, if applicable | 9-11 |
| Data sources/ measurement | 8* | For each variable of interest, give sources of data and details of methods of assessment (measurement). Describe comparability of assessment methods if there is more than one group | 9-11 |
| Bias | 9 | Describe any efforts to address potential sources of bias | 12-13 |
| Study size | 10 | Explain how the study size was arrived at | 12 |
| Quantitative variables | 11 | Explain how quantitative variables were handled in the analyses. If applicable, describe which groupings were chosen and why | 12-13 |
| Statistical methods | 12 | (*a*) Describe all statistical methods, including those used to control for confounding | 12-13 |
|  |  | (*b*) Describe any methods used to examine subgroups and interactions | 12-13 |
|  |  | (*c*) Explain how missing data were addressed | 12 |
|  |  | (*d*) If applicable, explain how loss to follow-up was addressed | NA |
|  |  | (*e*) Describe any sensitivity analyses | 12-13 |
| Results | | |  |
| Participants | 13* | (a) Report numbers of individuals at each stage of study—eg numbers potentially eligible, examined for eligibility, confirmed eligible, included in the study, completing follow-up, and analysed | 13 |
|  |  | (b) Give reasons for non-participation at each stage | 13 |
|  |  | (c) Consider use of a flow diagram | Figure 1 |
| Descriptive data | 14* | (a) Give characteristics of study participants (eg demographic, clinical, social) and information on exposures and potential confounders | 14-17 |
|  |  | (b) Indicate number of participants with missing data for each variable of interest | 14-22 |
|  |  | (c) Summarise follow-up time (eg, average and total amount) | 13-22 |
| Outcome data | 15* | Report numbers of outcome events or summary measures over time | 13-22 |
| Main results | 16 | (*a*) Give unadjusted estimates and, if applicable, confounder-adjusted estimates and their precision (eg, 95% confidence interval). Make clear which confounders were adjusted for and why they were included | 13-22 |
|  |  | (*b*) Report category boundaries when continuous variables were categorized | NA |
|  |  | (*c*) If relevant, consider translating estimates of relative risk into absolute risk for a meaningful time period | NA |
| Other analyses | 17 | Report other analyses done—eg analyses of subgroups and interactions, and sensitivity analyses | 14, 16-22, Tables S4-S6, Table S8 |
| Discussion | | |  |
| Key results | 18 | Summarise key results with reference to study objectives | 22-27 |
| Limitations | 19 | Discuss limitations of the study, taking into account sources of potential bias or imprecision. Discuss both direction and magnitude of any potential bias | 27 |
| Interpretation | 20 | Give a cautious overall interpretation of results considering objectives, limitations, multiplicity of analyses, results from similar studies, and other relevant evidence | 27 |
| Generalisability | 21 | Discuss the generalisability (external validity) of the study results | 27 |
| Other information | | |  |
| Funding | 22 | Give the source of funding and the role of the funders for the present study and, if applicable, for the original study on which the present article is based | 3, 30-31 |

**Table S2.** Participating study sites and recruitment details.

| **Site, Country** | **Hospital**  **type** | **No of beds** | **No of admissions** | **Confirmed AMI** | **Suspected AMI** | **SBO** |
| --- | --- | --- | --- | --- | --- | --- |
| Tartu University Hospital, Estonia | University | 962 | 26507 | 22 | 3 | 13 |
| North Estonia Medical Centre, Tallinn, Estonia | Regional | 1100 | 22200 | 16 | 0 | 11 |
| Northern State Medical University and City Hospital #1, Arkhangelsk, Russia | University | 1050 | 20873 | 27 | 7 | 5 |
| Universiti Sains Malaysia, Kubang Kerian, Kelantan, Malaysia | University | 900 | 32936 | 3 | 6 | 15 |
| Hospital Melaka, Malaysia | Local | 900 | 57993 | 10 | 0 | 0 |
| N.Kipshidze Central University Hospital, Tbilisi, Georgia | University | 350 | 10581 | 5 | 7 | 1 |
| Lucerne Cantonal Hospital, Lucerne, Switzerland | Regional | 850 | 26914 | 22 | 25 | 4 |
| Hadassah Medical Center and Faculty of Medicine, Hebrew University of Jerusalem, Israel | University | 1000 | 57214 | 22 | 5 | 12 |
| Universitätsklinikum Schleswig-Holstein, Campus Kiel, Germany | University | 1200 | 38296 | 34 | 24 | 0 |
| Hospital General San Martin de La Plata, Buenos Aires, Argentina | University | 325 | 9580 | 9 | 3 | 3 |
| University Hospital Centre Zagreb, Croatia | University | 1600 | 43387 | 4 | 2 | 2 |
| Virgen del Rocío University Hospital, Seville, Spain | University | 1200 | 30384 | 24 | 7 | 4 |
| Letterkenny University Hospital, Ireland | University | 350 | 17016 | 3 | 4 | 2 |
| Erciyes University Hospital Kayseri, Turkey | University | 1800 | 87752 | 11 | 1 | 0 |
| „Nicolae Testemitanu“ State University of Medicine and Pharmacy of the Republic of Moldova, Chisinau, Moldova | University | 620 | 24944 | 13 | 17 | 0 |
| Sanjay Gandhi Post Graduate Institute of Medical Sciences, Lucknow, India | University | 1200 | 29941 | 3 | 2 | 1 |
| Maulana Azad Medical College and Lok Nayak Hospital, New Delhi, India | University | 1800 | 62005 | 9 | 0 | 2 |
| Rabin Medical Center, University of Tel Aviv, Petah Tikva, Israel | University | 850 | 40035 | 14 | 4 | 8 |
| Fujian Provincial Hospital, China | Regional | 2120 | 39272 | 13 | 2 | 1 |
| Sarawak General Hospital, Malaysia | Regional | 765 | 38735 | 13 | 18 | 4 |
| Hospital Bintulu, Malaysia | Local | 332 | 2283 | 2 | 0 | 2 |
| Hospital Ampang, Malaysia | Local | 560 | 18798 | 2 | 1 | 9 |
| Queen Elisabeth Hospital, Kota Kinabalu, Sabah, Malaysia | Local | 800 | 35892 | 1 | 0 | 3 |
| Hospital Sultanah Nur Zahirah, Kuala Terengganu, Malaysia | Regional | 821 | 65894 | 2 | 0 | 1 |
| Division of General Surgery, University Hospital of Trieste ASUGI, Trieste, Italy | University | 500 | 13846 | 16 | 2 | 0 |
| Azienda Ospedaliera Universitaria Careggi, Firenze, Italy | University | 1400 | 32083 | 10 | 2 | 8 |
| Hospital Pengajar Universiti Putra, Malaysia | University | 400 | 5679 | 1 | 1 | 5 |
| Stavanger University Hospital, Norway | University | 650 | 23485 | 5 | 2 | 1 |
| AOU Citta della Salute e della Scienza, Turin, Italy | University | 2441 | 27295 | 8 | 3 | 1 |
| University Hospital North-Norway, Tromsø, Norway | University | 450 | 15837 | 8 | 1 | 0 |
| Royal Infirmary of Edinburgh, Scotland | University | 800 | 28705 | 8 | 10 | 10 |
| Intestinal Stroke Center, Department of Gastroenterology, IBD and Intestinal Failure, AP-HP. Nord, Beaujon Hospital, Paris Cité University, Paris, France | University | 1049 | 43699 | 78 | 0 | 0 |
| **Total** |  | **31145** | **1014224** | **418** | **159** | **128** |

**Table S3.** CT contrast enhancement protocols used for confirmation of the diagnosis of AMI.

|  | **All**  **(n=303)** | **Arterial (n=188)** | **Venous (n=69)** | **NOMI (n=29)** | **Other**  **(n=5)** | **Unclear (n=12)** |
| --- | --- | --- | --- | --- | --- | --- |
| Arterial + venous or delayed n (%) | 152 (50.2) | 98 (52.1) | 36 (52.2) | 11 (37.9) | 3 (60.0) | 4 (33.3) |
| Arterial only n (%) | 87 (28.7) | 68 (36.2) | 3 (4.3) | 12 (41.4) | 1 (20.0) | 3 (25.0) |
| Venous and/or delayed n (%) | 59 (19.5) | 19 (10.1) | 29 (42.0) | 6 (20.7) | 1 (20.0) | 4 (33.3) |
| No contrast n (%) | 5 (1.7) | 3 (1.6) | 1 (1.4) | - | - | 1 (8.3) |

NOMI – non-occlusive mesenteric ischaemia

**Table S4.** Baseline characteristics and hospital mortality of patients with confirmed AMI compared to those with suspected but not confirmed AMI and SBO. Full data including all sites are used for comparisons. Additionally, one site (Beaujon Hospital, Paris) was excluded from the main analysis (Table 2, main manuscript) and is presented separately.

|  | **No AMI**  **n = 159** | **SBO**  **n = 128** | **AMI all sites**  **n = 418** | **Paris**  **n = 78** | **P-value** | |
| --- | --- | --- | --- | --- | --- | --- |
|  |  |  |  |  | **No AMI vs. AMI *** | **SBO vs. AMI *** |
| **Demographics** | | | | | | |
| Ethnicity, n (%) (n=705) African | 1 (0.6) | 0 (0) | 3 (0.7) | 1 (1.3) |  |  |
| Arabian | 1 (0.6) | 3 (2.3) | 17 (4.1) | 6 (7.7) |  |  |
| Asian | 2 (1.3) | 10 (7.8) | 21 (5.0) | - |  |  |
| South Asian | 25 (15.7) | 20 (15.6) | 17 (4.1) | - |  |  |
| East Asian | 6 (3.8) | 12 (9.4) | 22 (5.3) | - |  |  |
| Caucasian | 114 (71.7) | 71 (55.5) | 307 (73.4) | 69 (88.5) |  |  |
| Hispanic | 8 (5.0) | 11 (8.6) | 18 (4.3) | - |  |  |
| Other | 2 (1.3) | 1 (0.8) | 13 (3.1) | 2 (2.6) | <0.001 | <0.001 |
| Gender (male), n (%) (n=705) | 88 (55.3) | 60 (46.9) | 241 (57.7) | 43 (55.1) | 0.637 | 0.002 |
| Age, median (range) (n=705) | 69 (23-97) | 70.5 (24-96) | 70 (18-99) | 67 (26-93) | 0.466 | 0.920 |
| BMI, kg/m^2^ (n=535) | 24.9  (22.9-27.7) | 24.2  (20.4-27.7) | 24.9  (21.8-28.2) | 24.1  (20.5-57.3) | 0.802 | 0.232 |
| **Chronic health conditions** | | | | | | |
| Disability ^a^, n (%) (n=661) | 43 (27.0) | 32 (25.0) | 83 (19.9) | 8 (10.3) | 0.088 | 0.006 |
| Smoking, n (%) (n=546) Current | 31 (19.5) | 20 (15.6) | 102 (24.4) | 35 (44.9) |  |  |
| Former | 26 (16.4) | 15 (11.7) | 40 (9.6) | - | 0.062 | 0.076 |
| Atrial fibrillation, n (%) (n=682) | 34 (21.4) | 24 (18.8) | 102 (24.4) | 9 (11.5) | 0.657 | 0.188 |
| Atherosclerosis, n (%) (n=667) | 61 (38.4) | 35 (27.3) | 151 (36.1) | 13 (16.7) | 0.489 | 0.067 |
| Arterial hypertension, n (%) (n=689) | 99 (62.3) | 70 (54.7) | 269 (64.4) | 44 (56.4) | 0.693 | 0.056 |
| Previous MI, n (%) (n=677) | 23 (14.5) | 10 (7.8) | 80 (19.1) | 3 (3.8) | 0.269 | 0.002 |
| Previous thromboembolic events, | 17 (10.7) | 7 (5.5) | 62 (14.8) | 2 (2.6) | 0.274 | 0.004 |
| n (%) (n=668) Arterial | 9 (52.9) | 3 (42.9) | 39 (63.9) | 2 (100.0) |  |  |
| Venous | 8 (47.1) | 4 (57.1) | 22 (36.1) | - | 0.416 | 0.415 |
| Charlson comorbidity index (n=651) | 4 (2-6) | 4 (2-5) | 4 (2-6) | 3 (2-4) | 0.873 | 0.022 |
| AMI suspected in ED, n (%) | 90 (56.6) | 79 (61.7) | 209 (50.0) | 74 (94.9) | 0.163 | 0.026 |
| AMI suspected later despite features present in ED, n (%) | 19 (11.9) | 19 (14.8) | 79 (18.9) | 4 (5.1) | 0.048 | 0.357 |
| AMI occurred later (admission for another diagnosis), n (%) | 50 (31.4) | 30 (23.4) | 130 (31.1) | - | 1 | 0.120 |
| Arrival to hospital, n (%) (n=378)  Self | 37 (41.1) | 36 (45.6) | 108 (51.7) | 64 (86.5) |  |  |
| Ambulance | 52 (57.8) | 42 (53.2) | 96 (45.9) | 9 (12.2) |  |  |
| Other | 1 (1.1) | 1 (1.3) | 5 (2.4) | 1 (1.4) | 0.182 | 0.579 |
| Time from beginning of symptoms to arrival to hospital, hours (n=454) | 20 (5-48) | 24 (8-48) | 24 (9-48) | 24 (24-48) | 0.639 | 0.851 |
|  | **No AMI** | **SBO** | **All AMI** | **Paris** | **No AMI vs. AMI** | **SBO vs. AMI** |
| **Initial diagnosis** | | | | | | |
| Time to suspicion of AMI ^#^, h (n=490) | 2.4 (1-7) | 2 (1-5) | 4.9 (1-12) | 6 (2-24) | <0.001 | <0.001 |
| Time from admission to suspicion of AMI in ED, hours (n=376) | 2 (1-4) | 2 (1-4) | 2 (1-6) | 6 (2-24) | 0.200 | 0.013 |
| Time from admission to suspicion of AMI if later, despite symptoms present in ED, hours (n=114) | 12 (3.6-108) | 9.6 (3-12) | 12 (12-16.1) | 24 (12-36) | 0.642 | 0.186 |
| Time from admission to suspicion of AMI, if occurred after admission due to another diagnosis, days (n=210) | 3 (1-8) | 1 (1-2.75) | 2 (1-8) | - | 0.419 | 0.015 |

| **Acute health conditions at admission/baseline** | | | | | | |
| --- | --- | --- | --- | --- | --- | --- |
| APACHE II, points (n=411) | 16 (8-22) | 10 (6-14) | 15 (9-23) | 7 (5.8-12) | 0.550 | <0.001 |
| SOFA, points (n=443) | 3 (1-8) | 2 (0-4) | 4 (2-9) | 2 (0-4) | 0.159 | <0.001 |
| New atrial fibrillation, n (%) (n=705) | 6 (3.8) | 4 (3.1) | 33 (7.9) | 0 | 0.095 | 0.070 |
| MV, n (%) (n=705) | 46 (28.9) | 45 (35.2) | 166 (39.7) | 12 (15.4) | 0.011 | <0.001 |
| Vasopressors, n (%) (n=705) | 30 (18.9) | 9 (7.0) | 112 (26.8) | 8 (10.3) | 0.052 | <0.001 |
| **Main symptoms of AMI** | | | | | | |
| Acute abdominal pain, n (%) | 126 (79.2) | 120 (93.8) | 336 (80.4) | 76 (97.4) | 0.816 | <0.001 |
| Diarrhea, n (%) | 23 (14.5) | 7 (5.5) | 73 (17.5) | 26 (33.3) | 0.453 | <0.001 |
| Bloody stool, n (%) | 13 (8.2) | 5 (3.9) | 39 (9.3) | 4 (5.1) | 0.747 | 0.062 |
| Shock, n (%) | 29 (18.2) | 5 (3.9) | 90 (21.5) | - | 0.421 | <0.001 |
| Abdominal distension, n (%) | 5 (3.1) | 4 (3.1) | 12 (2.9) | 1 (1.3) | 0.790 | 1 |
| Nausea/vomiting, n (%) | 5 (3.1) | 13 (10.2) | 42 (10.0) | 25 (32.1) | 0.006 | 1 |
| Absence of passage, n (%) | 8 (5.0) | 6 (4.7) | 6 (1.4) | - | 0.028 | 0.039 |
| No symptoms, n (%) | 4 (2.5) | 0 | 7 (1.7) | 1 (1.3) | 0.505 | 0.208 |
| **Laboratory results** | | | | | | |
| WBC, cells x10^9^ (n=681) | 12.7 (8-18.2) | 12.4 (8.2-16) | 16 (11.1-21) | 14 (9.4-21.0) | <0.001 | <0.001 |
| CRP, mg/L (n=540) | 45 (10-123) | 36 (6-112) | 100 (30-213) | 77 (29-182) | <0.001 | <0.001) |
| Creatinine, µmol/L (n=666) | 101 (75-146) | 90 (71-138) | 113 (78-190) | 71.5 (57.0-99.3) | 0.142 | 0.010 |
| eGFR, ml/min/1.73m^2^ (n=500) | 59 (32-85) | 60 (40-84) | 55 (27-94) | 90 (60.5-101.5) | 0.474 | 0.634 |
| ASAT, U/L (n=525) | 32 (21-118) | 24 (18-32) | 39 (24-82) | 29 (21.5-40.0) | 0.323 | <0.001 |
| Amylase, U/L (n=338) | 51 (28-116) | 67 (37-123) | 63 (35-132) | 35 (35-35) | 0.201 | 0.939 |
| Troponin T, ng/L (n=224) | 49 (11-240) | 30 (10-100) | 40 (13-134) | 22 (13-52) | 0.125 | 0.069 |
| pH (n=532) | 7.34 (7.21-7.4) | 7.38 (7.3-7.4) | 7.33 (7.23-7.4) | 7.4 (7.0-7.5) | 0.881 | <0.001 |
| BE (n=459) | -4.1 (-11.5; 0.2) | -0.8 (-5.5; 2) | -5.5 (-11; -0.5) | - | 0.585 | <0.001 |
| D-dimers, mg/L (n=153) | 6 (4-16) | 1.45 (0.34-5) | 5 (2-10) | 2 (1-5) | 0.331 | 0.002 |
| Lactate, mmol/L (n=561) | 2.95 (1.55-7.1) | 2.15 (1.5-3.4) | 3.06 (1.6-6.7) | 1.5 (1.1-2.5) | 0.929 | 0.001 |
| AMI subtype n (%) Occlusive arterial | NA | NA | 231 (55.3) | 52 (66.7) |  |  |
| Occlusive venous | NA | NA | 73 (17.5) | 25 (32.1) |  |  |
| NOMI | NA | NA | 55 (13.2) | - |  |  |
| Other/Unclear | NA | NA | 59 (14.1) | 1 (1.3) | NA | NA |
| Hospital mortality, n (%) | 42 (26.4 ) | 19 (14.8) | 205 (49.0) | 6 (7.7) | <0.001 | <0.001 |

Legend: (n=x) after the name of the variable always indicates number of patients included in the analysis for this specific variable. Data are presented in median (interquartile range) if not stated otherwise.

^a^ Disability was defined as need for any assistance in everyday life.

* AMI all sites is compared to other groups.

# All patients admitted due to AMI, pooled patients diagnosed in the ED and the ones in whom suspicion was raised beyond ED, despite features of AMI were present at admission (presented separately in the next two rows).

AMI – acute mesenteric ischaemia; APACHE – Acute Physiology and Chronic Health Evaluation; ASAT – aspartate aminotransferase; BE – base excess, BMI – body mass index; CRP – C-reactive protein; ED – emergency department; eGFR – estimated glomerular filtration rate; MI – myocardial infarction; MV – mechanical ventilation; NOMI – non-occlusive mesenteric ischaemia; SOFA – sequential organ failure assessment; WBC – white blood cell count.

**Table S5.** Comparison of patients admitted via ED to patients referred from other hospitals. Sensitivity analysis separating Beaujon Hospital, Paris from referred patients in other sites.

|  | **Admitted via ED (not referred) N=178** | **Referred Paris N=77** | **Referred**  **other sites**  **N=37** | **P-value** | |
| --- | --- | --- | --- | --- | --- |
|  |  |  |  | **ED vs. referred Paris** | **ED vs. referred**  **other sites** |
| **Demographics** | | | | | |
| Gender (male), n (%) (n=292) | 94 (52.8) | 43 (55.8) | 24 (64.8) | 0.683 | 0.207 |
| Age, median (range) (n=292) | 74 (21-99) | 67 (26-93) | 69 (40-92) | 0.021 | 0.258 |
| BMI, kg/m^2^ (n=218) | 24.6 (21.4-27.8) | 24.2 (20.5-27.3) | 24.2 (22.7-25.7) | 0.398 | 0.800 |
| **Chronic health conditions** | | | | | |
| Disability ^a^, n (%) (n=275) | 42 (23.6) | 8 (10.4) | 7 (18.9) | 0.015 | 0.666 |
| Smoking, n (%) (n=233) Current | 34 (19.1) | 34 (44.2) | 7 (18.9) |  |  |
| Former | 16 (9.0) | - | 5 (13.5) | <0.001 | 0.466 |
| Atrial fibrillation, n (%) (n=287) | 55 (30.9) | 9 (11.7) | 13 (35.1) | <0.001 | 0.696 |
| Atherosclerosis, n (%) (n=280) | 73 (41.0) | 13 (16.9) | 15 (40.5) | <0.001 | 1 |
| Arterial hypertension, n (%) (n=285) | 122 (68.5) | 43 (55.8) | 21 (56.8) | 0.030 | 0.235 |
| Previous MI, n (%) (n=282) | 43 (24.2) | 3 (3.9) | 6 (16.2) | <0.001 | 0.388 |
| Previous thromboembolic events, | 30 (16.9) | 2 (2.6) | 11 (29.7) | <0.001 | 0.106 |
| n (%) (n=276) Arterial | 18 (60.0) | 2 (100.0) | 5 (45.5) |  |  |
| Venous | 12 (40.0) | - | 6 (54.5) | 0.516 | 0.489 |
| Charlson comorbidity index (n=268) | 5 (3-6) | 3 (2-4) | 4 (2-7) | <0.001 | 0.922 |
| Anticoagulants, n (%) (n=277) | 36 (20.2) | 44 (57.1) | 11 (29.7) | <0.001 | 0.271 |
| Antiplatelets, n (%) (n=270) | 58 (32.6) | 14 (18.2) | 13 (35.1) | 0.006 | 1 |
| Statins n (%) (n=271) | 69 (38.8) | 8 (10.4) | 12 (32.4) | <0.001 | 0.449 |
| **Emergency department/baseline data (incl. data from referring hospital where available)** | | | | | |
| Arrival to hospital, n (%) (n=209) Self | 35 (33.3) | 64 (83.1) | 9 (24.3) |  |  |
| Ambulance | 69 (65.7) | 9 (11.7) | 18 (48.6) |  |  |
| Other | 1 (1.0) | 1 (1.3) | 3 (8.1) | <0.001 | 0.068 |
| Time from ED admission to suspicion of AMI (hours), (n=207) | 2 (1-4) | 6 (2-24) | 1 (1-4) | <0.001 | 0.176 |
| **Acute health conditions at admission/baseline** | | | | | |
| APACHE II, points (n=165) | 15.5 (10-20) | 7 (5-12) | 12 (8-14) | <0.001 | 0.313 |
| SOFA, points (n=197) | 4 (2-7) | 2 (0-4) | 3 (2-8) | <0.001 | 0.960 |
| New atrial fibrillation, n (%) (n=292) | 16 (9.0) | - | 4 (10.8) | 0.004 | 0.756 |
| Cardiac arrest, n (%) (n=292) | 3 (1.7) | - | - | 0.556 | 1 |
| Mechanical ventilation, n (%) (n=292) | 62 (34.8) | 12 (15.6) | 11 (29.7) | 0.002 | 0.703 |
| Vasopressors, n (%) (n=292) | 30 (16.9) | 8 (10.4) | 10 (27.0) | 0.250 | 0.165 |
| **Main symptoms of AMI** | | | | | |
| Acute abdominal pain, n (%) (n=292) | 164 (92.1) | 75 (97.4) | 34 (91.9) | 0.160 | 1 |
| Diarrhea, n (%) (n=292) | 32 (18.0) | 26 (33.8) | 6 (16.2) | 0.009 | 1 |
| Bloody stool, n (%) (n=292) | 18 (10.1) | 4 (5.2) | 6 (16.2) | 0.234 | 0.265 |
| Shock, n (%) (n=292) | 36 (20.2) | - | 7 (18.9) | <0.001 | 1 |
| Abdominal distension, n (%) | 1 (0.6) | 1 (1.3) | - | 0.514 | 1 |
| Nausea/vomiting, n (%) | 11 (6.2) | 25 (32.5) | 1 (2.7) | <0.001 | 0.696 |
| Absence of passage, n (%) | 2 (1.1) | - | - | 1 | 1 |
| No symptoms, n (%) (n=292) | 2 (1.1) | 1 (1.3) | - | 1 | 1 |
| **Laboratory results** | | | | | |
| WBC, cells x10^9^ (n=283) | 16 (11.2-20.4) | 14 (9-21) | 15.7 (12.9-18.2) | 0.263 | 0.871 |
| CRP, mg/L (n=237) | 92.5 (29-204) | 76 (24-166) | 111 (9-221) | 0.384 | 0.812 |
| Creatinine, µmol/L (n=272) | 114 (83-190) | 72 (57-100) | 104 (78-189) | <0.001 | 0.952 |
| eGFR, ml/min/1.73m^2^ (n=176) | 53 (22-79) | 89.5 (59-101) | 44 (28-64) | <0.001 | 0.383 |
| ASAT, U/L (n=241) | 36.5 (24-65) | 29 (21-39) | 28 (19-57) | 0.012 | 0.197 |
| Amylase, U/L (n=142) | 63 (36-130) | 35 (35-35) | 59 (32-152) | 0.360 | 0.965 |
| Troponin T, ng/L (n=122) | 31.5 (12-130) | 22 (13-52) | 38 (14-67) | 0.412 | 0.660 |
| pH (n=221) | 7.35 (7.25-7.4) | 7.4 (7-7.45) | 7.38 (7.23-7.44) | 0.432 | 0.338 |
| BE (n=156) | -5 (-9.3, -0.1) | - | -1.9 (-8,2) | NA | 0.117 |
| D-dimers, mg/L (n=89) | 7 (2-14) | 2 (1-5) | 20.5 (2-2420) | 0.001 | 0.466 |
| Lactate, mmol/L (n=250) | 3.6 (1.9-6.91) | 1.5 (1.17-2.5) | 3.02 (1.8-5.5) | <0.001 | 0.401 |
| Hospital mortality, n (%) | 93 (52.2) | 6 (7.8) | 14 (37.8) | <0.001 | 0.148 |

Legend: Data presented in median (IQR) if not stated otherwise.

^a^ Disability was defined as need for any assistance in everyday life.

(n=x) after the name of the variable always indicates number of patients included in the analysis for this specific variable. Data are presented in median (interquartile range) if not stated otherwise.

AMI – acute mesenteric ischaemia; APACHE – Acute Physiology and Chronic Health Evaluation; ASAT – aspartate aminotransferase; BE – base excess, BMI – body mass index; CRP – C-reactive protein; ED – emergency department; eGFR – estimated glomerular filtration rate; MI – myocardial infarction; NOMI – non-occlusive mesenteric ischaemia; SOFA – sequential organ failure assessment; WBC – white blood cell count.

**Table S6.** Comparison of baseline characteristics and outcome of patients with no delay in diagnosis to patients in whom diagnosis was delayed according to the assessment of the investigator.

|  | **Not delayed (n=78)** | **Delayed (n=75)** | **P-value** |
| --- | --- | --- | --- |
| Age, median (range) (n=153) | 75 (28-99) | 74 (21-96) | 0.798 |
| Atrial fibrillation, n (%) (n=150) | 25 (32.1) | 24 (32.0) | 1 |
| Previous myocardial infarction, n (%) (n=147) | 17 (21.8) | 21 (28.0) | 0.452 |
| Previous thromboembolic events, n (%) (n=141) | 12 (15.4) | 16 (21.3) | 0.400 |
| Anticoagulants, n (%) (n=145) | 13 (16.7) | 20 (26.7) | 0.166 |
| Antiplatelet drugs, n (%) (n=139) | 26 (33.3) | 23 (30.7) | 0.859 |
| Arrival to hospital, n (%) (n=100) Self | 22 (32.8) | 12 (36.4) |  |
| Ambulance | 44 (65.7) | 21 (63.6) | 0.882 |
| Arrival to hospital since beginning of symptoms, hours, (n=141) | 24 (8-72) | 20 (5-48) | 0.244 |
| Time from admission to suspicion of AMI, hours (n=153) | 2 (1-4) | 12 (2.4-12) | <0.001 |
| Time from admission to diagnosis, hours (n=141) | 3 (2-6) | 12 (6-12) | <0.001 |
| Type of ED pt was admitted n (%) (n=153 Surgical | 14 (17.9) | 24 (32.0) |  |
| Non-surgical | 15 (19.2) | 17 (22.7) |  |
| Mixed | 49 (62.8) | 34 (45.3) | 0.069 |
| APACHE II, points (n=93) | 15 (10-21) | 15.5 (9-22) | 0.788 |
| SOFA total, points (n=92) | 4 (2-7) | 3.5 (2-6) | 0.416 |
| Mechanical ventilation, n (%) (n=153) | 31 (24.8) | 19 (13.9) |  |
| Vasopressors, n (%) (n=153) | 11 (14.1) | 12 (16.0) | 0.823 |
| Acute abdominal pain, n (%) (n=153) | 71 (91.0) | 71 (94.7) | 0.534 |
| Diarrhea, n (%) (n=153) | 13 (16.7) | 14 (18.7) | 0.833 |
| Bloody stool, n (%) (n=153) | 7 (9.0) | 10 (13.3) | 0.447 |
| Shock, n (%) (n=153) | 19 (24.4) | 11 (14.7) | 0.156 |
| Abdominal distension, n (%) (n=153) | 0 | 0 | NA |
| Nausea/vomiting, n (%) (n=153) | 7 (9.0) | 2 (2.7) | 0.167 |
| Absence of passage, n (%) (n=153) | 1 (1.3) | 1 (1.3) | 1 |
| Other AMI sympt, n (%) (n=153) | 8 (10.3%) | 3 (4.0) | 0.210 |
| No symptoms, n (%) (n=153) | 1 (1.3) | 1 (1.3) | 1 |
| Speciality of doctor, n (%) (n=153) Surgery | 23 (29.5) | 22 (29.3) |  |
| Medicine | 0 | 10 (13.3) |  |
| Emergency medicine | 54 (69.2) | 38 (50.7) |  |
| Critical care | 1 (1.3) | 3 (4.0) | 0.001 |
| Time from admission to diagnosis, hours (n=88) | 3 (2-5) | 6 (3-10) | 0.006 |
| First radiographic study CT, n(%) (n=153) | 57 (73.1) | 44 (58.7) | 0.064 |
| Time from admission to CT-scan, hours (n=92) | 2 (1-5) | 4 (2-6) | 0.019 |
| Phases of CT- enhancement, n (%) (n=140) no contrast | 14 (19.2) | 7 (10.4) |  |
| portal venous/delayed phase | 9 (12.3) | 13 (19.4) |  |
| arterial | 15 (20.5) | 9 (13.4) |  |
| arterial + venous | 35 (47.9) | 38 (56.7) | 0.223 |
| AMI suspicion mentioned in the referral for the first radiographic study, n (%) (n=149) | 45 (57.7) | 26 (34.7) | 0.009 |
| Radiologist diagnosed AMI, n (%) (n=144) | 69 (88.5) | 53 (70.7) | 0.021 |
| Time between CT-scan and the response by radiologist, minutes (n=100) | 30 (15-45) | 30 (15-45) | 0.942 |
| AMI type, n (%) (n=153) Arterial | 52 (66.7) | 45 (60.0) |  |
| Venous | 17 (21.8) | 13 (17.3) |  |
| NOMI | 3 (3.8) | 7 (9.3) |  |
| Other | 6 (7.7) | 10 (13.3) | 0.331 |
| Acute on chronic mesenteric ischaemia, n (%) (n=139) | 10 (13.3) | 9 (12.7) | 0.945 |
| Lactate, mmol/L (n=129) | 3.98 (2.13-7.2) | 3.5 (1.8-6.8) | 0.562 |
| WBC, cells x10^9^ (n=148) | 15.6 (11-20.4) | 16.3 (11.5-20.7) | 0.408 |
| CRP, mg/L (n=121) | 68 (26-168) | 92.5 (23-193) | 0.816 |
| Creatinine, µmol/L (n=144) | 112 (82-187) | 114 (83-190) | 0.959 |
| eGFR, ml/min/1.73m^2^ (n=105) | 53.5 (23-75) | 53 (28-82) | 0.985 |
| ASATU/L (n=118) | 35 (23-63) | 38 (21-63) | 0.946 |
| Amylase, U/L (n=100) | 57 (37-113) | 63 (36-127) | 0.804 |
| Troponin-T, ng/L (n=54) | 26 (11-118) | 44 (16-130) | 0.215 |
| pH (n=124) | 7.35 (7.26-7.4) | 7.35 (7.26-7.41) | 0.922 |
| BE (n=118) | -4.2 (-9.25, -0.5) | -4.9 (-9.3, 0) | 0.963 |
| D-dimers, mg/L (n=40) | 7 (3-10) | 10 (3-42) | 0.119 |
| Time from presentation to any treatment, hours (n=128) | 4 (2-8) | 8 (6-20) | <0.001 |
| Time to revascularization from diagnosis of AMI, hours (n=34) | 5 (4-7) | 11 (4-14) | 0.170 |
| Time to intestinal resection from diagnosis of AMI, hours (n=57) | 3.5 (2-4) | 8 (2-13) | 0.020 |
| Hospital mortality, n (%) (n=153) | 42 (53.8) | 36 (48.0) | 0.519 |

Legend: Data presented in median (IQR) if not stated otherwise.

(n=x) after the name of the variable always indicates number of patients included in the analysis for this specific variable.

AMI – acute mesenteric ischaemia; APACHE – Acute Physiology and Chronic Health Evaluation; ASAT – aspartate aminotransferase; BE – base excess, BMI – body mass index; CRP – C-reactive protein; CT – computed tomography; ED – emergency department; eGFR – estimated glomerular filtration rate; SOFA – sequential organ failure assessment; WBC – white blood cell count.

**Table S7.** Systemic treatment before and after diagnosis of AMI.

|  | All (n=418) | Arterial (n=231) | Venous (n=73) | NOMI (n=55) | Other ^a^ (n=11) | Unclear (n=48) |
| --- | --- | --- | --- | --- | --- | --- |
| **Vasoactive treatment before diagnosis of AMI** | | | | | | |
| Norepinephine maximum dose before AMI (µg/kg/min) | 0.5  (0.2-1.2) | 0.5  (0.2-1.2) | 0.6  (0.02-0.8) | 0.52  (0.27-1.2) | 0.18  (0.18-0.18) | 0.39  (0.2-1) |
| Epinephrine maximum dose before AMI (µg/kg/min) | 0.0  (0.0-0.21) | 0.01  (0-0.22) | 0  (0-0) | 0.16  (0-0.46) | - | - |
| Vasopressin maximum dose before AMI (units/min) | 0.01  (0.0-0.03) | 0 (0-0.03) | 0 (0-0) | 0.03  (0-0.03) | - | 0.18  (0.03-0.33) |
| Other vasoactive drugs n (%) | 10 (2.4%) | 2 (0.9%) | 2 (2.7%) | 6 (10.9%) | - | - |
| Other vasoactives/inotropes used | Dobutamine, Methylene blue, Milrinone, Phenylephrine | | | | | |
| **Systemic treatment after diagnosis** | | | | | | |
| Vasopressors during 48h after initial treatment of AMI (n=295); n (%) | 152 (51.5%) | 93 (40.3%) | 9 (12.3%) | 24 (43.6%) | 5 (45.5%) | 21 (43.8%) |
| Norepinephrine; n (%) | 144 (34.4) | 87 (37.7) | 9 (12.3) | 24 (43.6) | 4 (36.4) | 20 (41.7) |
| Epinephrine; n (%) | 18 (4.3) | 10 (4.3) | 1 (1.4) | 4 (7.3) | 1 (9.1) | 2 (4.2) |
| Vasopressin; n (%) | 28 (6.7) | 15 (6.5) | - | 8 (14.5) | - | 5 (10.4) |
| Other vasoactive drugs; n (%) | 7 (1.7) | 4 (1.7) | 2 (2.7) | 1 (1.8) | - | - |
| Other vasoactives/inotropes used | Dobutamine, Dopamine, Milrinone, Phenylephrine | | | | | |
| Norepinephrine maximum dose (µg/kg/min) | 0.4  (0.18-1.21) | 0.4  (0.16-1.4) | 0.5  (0.07-0.6) | 0.35  (0.2-0.47) | 0.15  (0.1-7.5) | 0.9  (0.24-1.2) |
| Epinephrine maximum dose (µg/kg/min) | 0.2  (0.12-0.45) | 0.3  (0.13-0.44) | 0.2  (0.2-0.2) | 0.14  (0.03-0.2) | - | 1.6  (0.2-3) |
| Vasopressin maximum dose (units/min) | 2.0  (0.03-2.38) | 1.2  (0.02-2) | - | 2  (0.03-3) | - | 0.05  (0.03-2.5) |
| Total IV fluids during 48 hours after initial treatment (L) | 5.0  (2.8-6.6) | 4.8  (3.0-6.8) | 5.0  (3.0-6.3) | 5.3  (3.5-7.4) | 6.4  6.0-13.2) | 4.0  (1.9-5.5) |
| Maximum cumulative fluid balance during 48 hours after treatment (L) | 2.1  (1.0-4.0) | 2.8  (1.1-4.2) | 1.1  (0-2.7) | 2.2  (1.1-4.6) | 1.0  (1.0-5.4) | 1.4  (0.8-2.4) |
| Mechanical ventilation n=296; n (%) | 176 (59.5%) | 98 (52.7%) | 21 (63.6%) | 24 (82.8%) | 8 (88.9%) | 25 (64.1%) |
| Renal replacement therapy; n (%) | 41 (13.9%) | 18 (9.7%) | 2 (6.1%) | 14 (48.3%) | 3 (33.3%) | 4 (10.3%) |
| **Nutrition during 0-24h after initial treatment (n (%)** | | | | | | |
| None; n (%) | 201 (48.1) | 123 (53.2) | 21 (28.8) | 18 (32.7) | 6 (54.5) | 33 (68.8) |
| Oral; n (%) | 8 (1.9) | 5 (2.2) | 2 (2.7) | 1 (1.8) | - | - |
| Enteral; n (%) | 16 (3.8) | 11 (4.8) | - | 3 (5.5) | - | 2 (4.2) |
| Parenteral; n (%) | 77 (18.4) | 51 (22.1) | 10 (13.7) | 9 (16.4) | 3 (27.3) | 4 (8.3) |
| **Nutrition during 24-48h after initial treatment (n (%)** | | | | | | |
| None; n (%) | 162 (38.8) | 99 (42.9) | 17 (23.2) | 17 (30.9) | 4 (36.4) | 25 (52.1) |
| Oral; n (%) | 26 (6.2) | 17 (7.4) | 4 (5.5) | 1 (1.8) | 1 (9.1) | 3 (6.3) |
| Enteral; n (%) | 24 (6.5) | 16 (6.9) | 2 (2.7) | 3 (35.5) | - | 3 (6.3) |
| Parenteral; n (%) | 92 (22) | 59 (25.5) | 11 (15.1) | 9 (16.4) | 4 (36.4) | 9 (18.8) |
| **Nutrition during 48-72h after initial treatment (n (%)** | | | | | | |
| None; n (%) | 127 (55) | 75 (32.5) | 13 (17.8) | 15 (45.1) | 3 (27.3) | 21 (43.8) |
| Oral; n (%) | 45 (10.8) | 31 (13.4) | 6 (8.2) | 2 (3.6) | 1 (9.1) | 5 (10.4) |
| Enteral; n (%) | 34 (8.1) | 21 (9.1) | 3 (4.1) | 5 (9.1) | - | 5 (10.4) |
| Parenteral; n (%) | 105 (25.1) | 69 (29.9) | 12 (16.4) | 10 (18.1) | 5 (45.5) | 9 (18.8) |
| Nutrition start day; | 3 (1-6) | 3 (1-6) | 4 (1-6) | 4 (1-8) | 6 (2-15) | 1.5 (0-3) |
| Total calories within 48 hours | 0 (0-0) | 0 (0-0) | 0 (0-0) | 0 (0-0) | 0 (0-0) | 0 (0-0) |
| Total calories within 7 days | 0  (0-1200) | 0  (0-1300) | 700  (400-1200) | 0  (0-1200) | 0  (0-0) | 0  (0-20) |

Legend: Data presented in median (IQR) if not stated otherwise

^a^ Other: included specific mechanisms such as dissection, bowel distortion, mechanical devices for cardiac support and abdominal compartment syndrome

NOMI – non-occlusive mesenteric ischaemia

**Table S8.** Comparison of baseline characteristics of patients with active treatment vs. end-of-life care without an attempt of curative treatment.

| **Management**  **Variable** | | | **Active treatment n = 372** | | **End-of-life care**  **n = 46** | | **P-value** |
| --- | --- | --- | --- | --- | --- | --- | --- |
| **Demographics** | | | | | | |  |
| Gender (male), n (%) (n=418) | | | 218 (58.6) | | 23 (50.0) | | 0.273 |
| Age, median (range) (n=418) | | | 69 (18-94) | | 76 (18-99) | | 0.002 |
| BMI, kg/m^2^ (n=319) | | | 25.1 (22-28.3) | | 21.4 (20-26.1) | | 0.010 |
| **Chronic health conditions** | | | | | |  |  |
| Disability ^a^, n(%) (n=418) | | | 70 (18.8) | | 13 (28.2) | | 0.104 |
| Previous myocardial infarction, n (%) (n=418) | | | 70 (18.8) | | 10 (21.7) | | 0.547 |
| Previous thromboembolic events, n (%) (n=418) | | | 55 (14.8) | | 7 (15.2) | | 0.824 |
| Previous thromboembolic events, n (%) (n=62) Arterial | | | 35 (63.6) | | 4 (57.1) | |  |
| Venous | | | 20 (36.4) | | 2 (28.6) | | 1 |
| Artificial heart valve, n (%) (n=418) | | | 11 (3.0) | | 2 (4.3) | | 0.643 |
| Charlson comorbidity index, (n=383) | | | 4 (2-6) | | 6 (3-7) | | 0.005 |
| Anticoagulants, n(%) (n=418) | | | 99 (26.6) | | 11 (23.9) | | 1 |
| Antiplatelet drugs, n(%) (n=418) | | | 108 (29.0) | | 15 (32.6) | | 0.266 |
| Statins (n=418) | | | 119 (32.0) | | 15 (32.6) | | 0.469 |
| **Emergency department/baseline data** | | | | | |  |  |
| AMI was the reason of admission, n (%) (n=418) | | | 186 (50.0) | | 23 (50.0) | |  |
| AMI suspected later, despite features present in ED | | | 74 (19.9) | | 5 (10.9) | |  |
| AMI occurred during hospital stay | | | 112 (30.1) | | 18 (39.1) | | 0.246 |
| Arrival to hospital, n (%) (n=209) Self | | | 104 (55.9) | | 4 (17.4) | |  |
| Ambulance | | | 77 (41.4) | | 19 (82.6) | |  |
| Other | | | 5 (2.7) | | - | | 0.001 |
| Time from admission to suspicion of AMI, hours (n=286) | | | 5.5 (2-12) | | 2 (1-5.6) | | 0.013 |
| Time from admission to suspicion of AMI when occurred during hospital stay, days (n=130) | | | 3 (1-12) | | 5.5 (1-11) | | 0.355 |
| **Location of patient at the time of suspicion of AMI** | | | | | | |  |
| Type of ED, n (%) (n=288) Surgical ED | | | 67 (25.8) | | 4 (14.3) | |  |
| Non-surgical ED | | | 64 (24.6) | | 9 (32.1) | |  |
| Mixed ED | | | 129 (49.6) | | 15 (53.6) | | 0.376 |
| AMI recognized/occurred in ward, n (%) (n=209) Surgical | | | 66 (35.5) | | 2 (8.7) | |  |
| Medical | | | 29 (15.6) | | 5 (21.7) | |  |
| ICU | | | 57 (30.6) | | 10 (43.4) | |  |
| IMC/HDU | | | 12 (6.5) | | - | |  |
| Other | | | 22 (11.8) | | 6 (26.1) | | 0.018 |
| **Acute health conditions at admission/baseline** | | | | | | |  |
| APACHE II, points (n=260) | | | 14 (8-21) | | 25 (17-27.5) | | <0.001 |
| SOFA total, points (n=294) | | | 3.5 (1-8) | | 6.5 (4-10) | | 0.001 |
| New atrial fibrillation, n (%) (n=418) | | | 29 (7.8) | | 4 (8.7) | | 0.773 |
| Cardiac arrest, n (%) (n=418) | | | 8 (2.2) | | 6 (13.0) | | 0.002 |
| Mechanical ventilation, n (%) (n=418) | | | 144 (38.7) | | 22 (47.8) | | 0.265 |
| Vasopressors before diagnosis, n (%) (n=418) | | | 94 (25.3) | | 18 (39.1) | | 0.053 |
| Norepinephrine dose, µg/kg/min (n=96) | | | 0.5 (0.19-1.2) | | 0.83 (0.5-1.2) | | 0.169 |
| Epinephrine dose, µg/kg/min (n=23) | | | 0 (0-0.16) | | 0.21 (0.02-0.22) | | 0.060 |
| Vasopressin dose, U/min (n=28) | | | 0 (0-0.03) | | 0.033 (0.03-0.05) | | 0.059 |
| Intra abdominal pressure, mmHg (n=36) | | | 14 (12-17) | | 14 (13-14.5) | | 0.729 |
| **Symptoms of AMI** | | | | | | | |
| Acute abdominal pain, n (%) (n=418) | | | 306 (82.3) | | 30 (65.2) | | 0.010 |
| Diarrhea, n (%) (n=418) | | | 68 (18.3) | | 5 (10.9) | | 0.302 |
| Bloody stool, n (%) (n=418) | | | 36 (9.7) | | 3 (6.5) | | 0.787 |
| Shock, n (%) (n=418) | | | 74 (19.9) | | 16 (34.8) | | 0.034 |
| Abdominal distension | | | 11 (3.0) | | 1 (2.2) | | 1 |
| Nausea/vomiting | | | 39 (19.5) | | 3 (6.5) | | 0.602 |
| Absence of passage | | | 2 (0.5) | | 4 (8.7) | | 0.002 |
| Other factors (eg. Lactate, systemic infection, IAH) | | | 12 (3.2) | | 3 (6.5) | | 0.209 |
| No symptoms suggesting AMI, n (%) (n=418) | | | 5 (1.3) | | 2 (4.3) | | 0.174 |
| **Acute conditions with known risk of AMI** | | | | | |  |  |
| Cardiac surgery, n (%) (n=209) | | | 8 (4.3) | | 2 (8.7) | | 0.303 |
| Aortic surgery, n (%) (n=209) | | | 12 (6.5) | | 1 (4.3) | | 1 |
| Embolisation, n (%) (n=130) | | | 3 (3.7) | | - | | 1 |
| Shock with high-dose vasopressors, n (%) (n=130) | | | 46 (41.1) | | 10 (43.5) | | 0.308 |
| **Laboratory results** |  |  | |  | |  |  |
| WBC, cells x10^9^ (n=404) | | | 16 (11.2-21) | | 16.5 (11-21) | | 0.805 |
| CRP, mg/L (n=339) | | | 100 (30-215) | | 112 (47-192) | | 0.518 |
| Creatinine, µmol/L (n=393) | | | 109 (76-179) | | 162 (102-249) | | 0.001 |
| eGFR, ml/min/1.73m^2^ (n=259) | | | 53 (27-85) | | 30 (17-53) | | 0.001 |
| ASAT, U/L (n=338) | | | 37 (22-72) | | 78 (48-322) | | <0.001 |
| Amylase, U/L (n=198) | | | 62 (35-132) | | 69 (48-148) | | 0.574 |
| Troponin T, ng/L (n=160) | | | 30.5 (13-110) | | 114 (45-315) | | 0.002 |
| pH (n=322) | | | 7.34 (7.24-7.4) | | 7.23 (7.08-7.3) | | <0.001 |
| BE (n=256) | | | -4.4 (-9, 0) | | -13.1 (-20, -6.2) | | <0.001 |
| D-dimers, mg/L (n=119) | | | 5 (2-9) | | 19 (4.7-43) | | 0.007 |
| Lactate 0-12h before diagnosis of AMI, mmol/L (n=349) | | | 3.66 (1.5-6) | | 6.9 (4.3-10.5) | | <0.001 |
| Lactate 12-24h before diagnosis of AMI, mmol/L (n=195) | | | 2.2 (1.4-3.9) | | 4.8 (2.7-6.1) | | 0.016 |
| **AMI type** | | | | | | | |
| Arterial | | | 205 (55.1) | | 26 (56.5) | | 0.003 |
| Venous | | | 72(19.4) | | 1 (2.2) | |  |
| NOMI | | | 47(12.6) | | 8 (17.4) | |  |
| Other/Unclear | | | 48 (12.9) | | 11 (23.9) | |  |

Legend: Data presented in median (IQR) if not stated otherwise.

^a^ Disability was defined as need for any assistance in everyday life.

(n=x) after the name of the variable always indicates number of patients included in the analysis for this specific variable.

AMI – acute mesenteric ischaemia; APACHE – Acute Physiology and Chronic Health Evaluation; ASAT – aspartate aminotransferase; BE – base excess, BMI – body mass index; CRP – C-reactive protein; ED – emergency department; eGFR – estimated glomerular filtration rate; NOMI – non-occlusive mesenteric ischaemia; SOFA – sequential organ failure assessment; WBC – white blood cell count.
